# Kv7 Channels as an Important Contributor to Alcohol-Induced Modulation of Neuronal Excitability in Neonatal Rat Superior Cervical Ganglion

**DOI:** 10.3390/cells14211723

**Published:** 2025-11-01

**Authors:** Da-Jeong Jeong, Jin-Nyeong Woo, Tery Yun, Myungin Baek, Byung-Chang Suh

**Affiliations:** Department of Brain Sciences, Daegu Gyeongbuk Institute of Science and Technology (DGIST), Daegu 42988, Republic of Korea; dajeong@dgist.ac.kr (D.-J.J.); tcc03120@dgist.ac.kr (J.-N.W.); tery0520@dgist.ac.kr (T.Y.); bmi008@dgist.ac.kr (M.B.)

**Keywords:** alcohol, neuronal excitability, Na_v_ channel, Kv7 channel, superior cervical ganglion

## Abstract

**Highlights:**

**What are the main findings?**

- Neuronal excitability changes induced by alcohols are determined by Kv7 modulation rather than sodium inhibition in rat neonatal SCG neurons.
- Short-chain alcohols enhance excitability through Kv7 inhibition, while long-chain alcohols reduce excitability via Kv7 activation.

**What is the implication of the main finding?**

- Kv7 channels represent an important target for n-alcohols in the regulation of neuronal excitability.
- The chain length-dependent modulation of Kv7 channels by alcohols provides mechanistic insights and guidance for Kv7 channel-targeted drug development.

**Abstract:**

Normal alcohols (*n*-alcohols) exhibit contrasting effects on neuronal excitability; specifically, ethanol enhances neuronal firing, while hexanol suppresses it. Both compounds are known to inhibit sodium currents, yet the mechanisms behind their differing effects remain unclear. Our previous studies demonstrated that Kv7 channels are modulated differently by alcohol chain length, prompting investigation into their role in these contrasting effects. We conducted whole-cell patch clamp recordings on neonatal (P5-P7) rat superior cervical ganglion neurons to assess alcohol impacts on action potential firing and ionic currents, utilizing tetrodotoxin (TTX), XE991, and retigabine (RTG). Ethanol (100 mM) increased action potential frequency, whereas hexanol (3 mM) decreased it, despite both inhibiting sodium currents by 12% and 45%, respectively. Notably, ethanol inhibited Kv7 currents by 16%, while hexanol enhanced them by 29%. TTX alone did not affect firing frequency until sodium current inhibition exceeded 76%, indicating moderate sodium channel blockade cannot fully explain the effects of alcohol. XE991 increased firing frequency and depolarized the resting membrane potential, while retigabine produced opposite effects. The combination of TTX with Kv7 modulators replicated the effects observed with each alcohol. These findings suggest Kv7 channel modulation plays an important role in the chain length-dependent effects of alcohol on neuronal excitability.

## 1. Introduction

Normal alcohols (*n*-alcohols) are organic psychoactive substances that exert sedative and anesthetic effects on both the central and peripheral nervous systems [1,2,3,4]. Previous studies have suggested that alcohol reduces neuronal firing primarily through the inhibition of sodium current [5,6,7,8]. However, ethanol is also known to modulate several other ion channels and neurotransmitter systems, including NMDA [9,10], GABA_A_ [11,12] and glycine receptors [13,14], indicating that its actions are multifaceted rather than limited to sodium channel inhibition. Therefore, this sodium channel-centric perspective does not adequately explain the contrasting effects on action potentials (APs) induced by *n*-alcohols of varying chain lengths, particularly the differences observed between short-chain ethanol (C2) and long-chain hexanol (C6). This discrepancy indicates that additional mechanisms may also play a role in regulating neuronal firing and influencing AP characteristics.

One potential mechanism involves Kv7 (KCNQ) channels, a subfamily of voltage-gated potassium channels responsible for generating the neuronal M-current. Kv7 channels are crucial regulators of neuronal firing due to their activation at subthreshold potentials and their non-inactivating properties [15,16,17,18]. The functional significance of Kv7 channels is underscored by findings that a single mutation reducing Kv7 current by approximately 25% leads to an abnormal increase in neuronal firing [19]. Given their critical role in controlling neuronal excitability, Kv7 channels have emerged as promising targets for treating hyperexcitability disorders such as epilepsy and chronic pain [20,21].

Recent studies have demonstrated that ethanol inhibits Kv7 channels [22]. This finding is also conserved in Drosophila, linking the inhibition of Kv7 channels to changes in neural activity and behavioral outcomes [23]. Additionally, mechanistic studies have shown that ethanol reduces the open probability of the Kv7.2 channels by decreasing the sensitivity to phosphatidylinositol 4,5-bisphosphate (PIP_2_) [24]. Building on these findings, our previous research demonstrated that *n*-alcohols regulate Kv7 currents in superior cervical ganglion (SCG) neurons in a chain length-dependent manner [25]. Specifically, long-chain *n*-alcohols activate Kv7 currents through the tryptophan residue at position 236 in the S5 segment of the Kv7.2 channel. However, whether these opposing effects on Kv7 channels translate into corresponding changes in AP firing remains unexplored. While ethanol is the most clinically relevant alcohol, studying both short- and long-chain *n*-alcohol provides critical mechanistic insight by distinguishing ethanol specific effects from general chain length dependent actions. Moreover, the simple, linear structure of *n*-alcohols makes them ideal model compounds for systematically analyzing the fundamental biophysical determinants of Kv7 channel modulation.

Based on these observations, we hypothesized that *n*-alcohols modulate neuronal firing through a dual-target mechanism that simultaneously affects sodium and Kv7 channels. To test this hypothesis, we compared the effects of ethanol and hexanol with selective pharmacological modulators that mimic the degree of sodium and Kv7 channel modulation induced by these alcohols. By applying these modulators individually and in combination, we demonstrate that while both alcohols inhibit sodium channels, differential Kv7 channel modulation is an important contributor to their contrasting effects on neuronal firing. These results provide a new mechanistic framework for understanding the impact of alcohol on the nervous system and highlight the potential of Kv7 channels as therapeutic targets for modulating neuronal excitability.

## 2. Materials and Methods

### 2.1. SCG Neuronal Preparations

SCG neurons were cultured using the previously described protocol [26]. In brief, the isolated ganglia were placed in RPMI 1640 medium (Gibco, Thermo Fisher Scientific, Waltham, MA, USA), treated with 0.25% trypsin, and incubated for 20 min at 37 °C. Following the enzyme treatment, the ganglia were suspended in RPMI 1640 medium containing 10% heat-inactivated horse serum and 5% fetal bovine serum to stop digestion. Then, they were resuspended in RPMI 1640 medium containing 1% heat-inactivated horse serum, 100 ng/mL 2.5 S nerve growth factor (NGF), 0.4% penicillin/streptomycin, and 0.4% uridine/5-fluorodeoxyuridine. The neuronal cells were plated on chips coated with 0.1 mg/mL poly-L-lysine in borate buffer and incubated at 37 °C in 5% CO_2_. Plated neuronal cells were studied in electrophysiological recording within 2 days after plating.

### 2.2. Animals

Sprague-Dawley rats were purchased from SamTako Bio Korea (Osan, Republic of Korea). All animal procedures were approved by the Institutional Animal Care and Use Committee at Daegu Gyeongbuk Institute of Science and Technology (Approval Number: DGIST-IACUC-21091703-0001 and DGIST-IACUC-24042309-0000), including the use of carbon dioxide (CO_2_) euthanasia. Animals were immediately removed from the chamber once movement ceased to minimize any potential pain or discomfort. Neonates (5 to 7 days after birth) were used to prepare SCG.

### 2.3. Cell Culture and Transfection

HEK293T cells were cultured in Dulbecco’s Modified Eagle Medium (DMEM) (HyClone, Thermo Fisher Scientific) supplemented with 10% fetal bovine serum (HyClone, Thermo Fisher Scientific) and 0.2% penicillin/streptomycin (HyClone, Thermo Fisher Scientific) in 100 mm culture dishes at 37 °C with 5% CO_2_. When the confluency of cells reached about 80%, they were subcultured using Dulbecco’s Phosphate-Buffered Saline (DPBS) (Gibco, Thermo Fisher Scientific) to detach the cells. Cells were cultured in 35 mm culture dishes according to the experimental schedule for transfection. Kv7.2 (GenBank accession number AF110020) and Kv7.3 (accession number NM_004519) channels were transiently transfected at a 1:1 molar ratio with 800 ng M1R (accession number NM_080773) and 200 ng pEGFP-N1 vector as a transfection marker to measure the Kv7.2/7.3 current. PIP 5-kinase type Iγ (500 ng, PIPKIγ; provided by Y. Aikawa and T. F. Martin, University of Wisconsin, Madison, WI, USA) was cotransfected to increase the plasma membrane PI(4,5)P_2_ level. Lipofectamine 2000 (Invitrogen, Waltham, MA, USA) was used for transfection when the confluency of cells reached 70–80% in a 35 mm culture dish. The transfected HEK293T cells were plated onto coverslip chips coated with 0.1 mg/mL poly-L-lysine (Sigma-Aldrich, St. Louis, MO, USA) 12 to 18 h after transfection. Plated cells were studied in electrophysiological recording 8 to 16 h after plating.

### 2.4. Electrophysiological Recording

SCG neuronal cells were whole-cell clamped at room temperature (22–25 °C) using a HEKA EPC-10 amplifier with Pulse software (HEKA Elektronik, Pfalz, Germany, https://www.heka.com). The electrodes were pulled from glass micropipette capillaries using a P-97 micropipette puller (Sutter Instrument, Novato, CA, USA) that had a resistance of 2–5 MΩ. The whole-cell access resistance was 2–6 MΩ, and series-resistance errors were compensated by 60%. For recording Kv7 current, cells were held at −20 mV and applied a 500 ms hyperpolarizing step to −55 mV every 4 s. In all voltage protocols, the holding potential was −80 mV. Activation Na^+^ currents were recorded with a membrane holding potential of −100 mV and applying 25 ms test pulses from −70 mV to +50 mV with 10 mV increment. For action potential recording, current-clamp mode was used. The current injection was held at 0 and action potential elicited by a 100 pA for 1 s.

### 2.5. Solution and Materials

The extracellular solution used for Kv7 currents, Kv7.2/7.3 currents and action potential recording contained 160 mM NaCl, 2.5 mM KCl, 2 mM CaCl_2_, 1 mM MgCl_2_, 10 mM HEPES, and 8 mM glucose, adjusted to pH 7.4 with NaOH. The intracellular solution of pipette consisted of 175 mM KCl, 5 mM MgCl_2_, 5 mM HEPES, 0.1 mM BAPTA, 3 mM Na_2_ATP, and 0.1 mM Na_3_GTP, adjusted to pH 7.2 with KOH. To minimize space-clamping error and record consistent Na^+^ peak current, the Na^+^ gradient was reversed [27]. In inverse Na^+^ gradient, the extracellular solution consisted of 2 mM NaCl, 3 mM KCl, 2 mM CaCl_2_, 2 mM MgCl_2_, 10 mM HEPES, 10 mM glucose and 244 mM sucrose, adjusted to pH 7.4 with TEAOH. The receptor antagonists were added just before the experiment to block various receptors expressed on neuronal cells. The receptor antagonists were as follows: 4 μM CNQX, 25 μM DL-AP5, 100 μM PTX, 30 mM TEACl, 4 mM 4-AP and 50 μM CdCl_2_. The intracellular solution used for inverse Na^+^ current recording contained 140 mM NaCl, 20 mM CsCl, 20 mM CsF, 2 mM MgCl_2_, 10 mM HEPES, 0.1 mM BAPTA-K4, 3 mM Na_2_ATP and 0.1 mM Na_3_GTP, adjusted to pH 7.2 with CsOH. Ethanol (EtOH) and butanol (BuOH) were purchased from Merck (Darmstadt, Germany), other *n*-alcohols used in this study including hexanol (HeOH) were purchased from Sigma-Aldrich. Retigabine (Cat#: 90221), PF-05089771 (Cat#: PZ0311), XE991 (Cat#: X2254) and Oxo-M (Cat#: O100) were purchased from Sigma-Aldrich. Tetrodotoxin citrate (TTX, Cat#: T-550) were purchased from Alomones labs. All compounds including *n*-alcohols were applied via a bath perfusion system for approximately 40 s, followed by washout with control external solution. They were stored as stocks in DMSO or distilled water (only TTX) and diluted to working concentrations in extracellular solution on each experimental day.

### 2.6. Reverse Transcription-PCR

Rat SCG total RNA was extracted from the whole SCG from Sprague-Dawley rats (P5−P7) by homogenization in QIAzol Lysis Reagent (QIAGEN, Venlo, The Netherlands). Total RNA from SCG were reverse transcribed using the TOPscript cDNA Synthesis kit (Enzynomics, Daejeon, Republic of Korea) according to the manufacturer’s instructions. Sequence-specific oligonucleotide primers were designed to all subtypes of the Na_V_ gene family. The primer sequences were as follows (5′→3′). Na_V_1.1, forward: gacaatgagtaaccctcctgact; reverse: gttctgtctcagtgtgtttgctc. Na_V_1.2, forward: catcttcggctcattcttcacc; reverse: ccagagtcaggagatgaccaac. Na_V_1.3, forward: cggctcaaagaaacctcagaag; reverse: gtggtggtgattctctcgattg. Na_V_1.4, forward: ggacctggatccttactacagtg; reverse: tacaccttcacggggatctatac. Na_V_1.5, forward:cagcagaagaaaaagttagggggccaggatatcttcatgac; reverse: gagacagatgaccagag-ccctgagaaggtcaacatcttg. Na_V_1.6, forward: catctgcagaaagatggcctcc; reverse: gaggagggcagaagggaaag. Na_V_1.7, forward: ggataggatcagtggttatggcag; reverse: caagttaatgtagactctgggaaaggg. Na_V_1.8, forward: gtagacatggagaagagggacag; reverse: gaagtatctgatctgggagtgct. Na_V_1.9, forward: gaggtagaggacgatgtggaata; reverse: acagagtcctcgaaagaagtctg. β-actin, forward: gggaaatcgtgcgtgacattaaag; reverse: cggatgtcaacgtcacacttc. Expression of the target gene was normalized to the housekeeping gene β-actin. Images were analyzed by Image J software (v1.52a).

### 2.7. Single-Cell RNA Sequencing Data Analysis

Single-cell RNA Sequencing Data Processing: Publicly available Drop-Seq data from the SCG of adult mice [28] were reanalyzed. Quality control of single-cell RNA-seq data, as well as data integration and analysis, were performed as described in the Supplementary Methods.

Cluster Annotation and Pseudo-bulk Analysis: Clusters were annotated to known cell types based on previously described signature genes [28]. For sympathetic neurons, pseudo-bulk expression profiles for genes of interest (Actb, Scn1a–Scn11a) were computed per sample using AggregateExpression function (Seurat version 5.3.0) on raw counts. Gene expression levels were normalized to Actb expression. A bar plot was generated using GraphPad Prism 7 (GraphPad Software).

### 2.8. Data Analysis and Statistical Analysis

All data were analyzed using IGOR Pro-6.34, Excel 2016 (Microsoft), MATLAB (R2022b) and GraphPad Prism 7 (GraphPad Software). Statistics in text or figure were expressed as mean ± SEM. Statistical analysis for differences between two groups was made by paired or unpaired Student’s *t*-tests. One-way analysis of variance (ANOVA) was carried out to compare multiple groups. Depending on the variance, ANOVA was followed by Tukey’s HSD post hoc test. The concentration-response curves were fitted by the Hill equation and data for G/G_max_ curves were fitted by the Boltzmann equation. The AP threshold was identified as the point where dV/dt showed a rapid increase [29]. Various studies have defined the AP threshold as the first point where dV/dt exceeds 10 to 40 mV/ms [30,31,32,33,34]. Since the purpose of threshold detection is to capture the actual onset of the AP, we set the AP threshold at the point where dV/dt exceeded 30 mV/ms to avoid erroneous threshold identification caused by the gradual dV/dt changes during the pre-AP depolarizing phase. The analysis was restricted to points within the current injection period only. The time at which the AP threshold was first reached was defined as the threshold time. AP threshold and threshold time measurements were performed using custom scripts in MATLAB. For all analyses, *p* value less than 0.05 was considered significant (*, *p* < 0.05; **, *p* < 0.01; and ***, *p* < 0.001).

## 3. Results

### 3.1. n-Alcohols Modulate Neuronal Excitability in SCG Neurons

**Figure 1:**
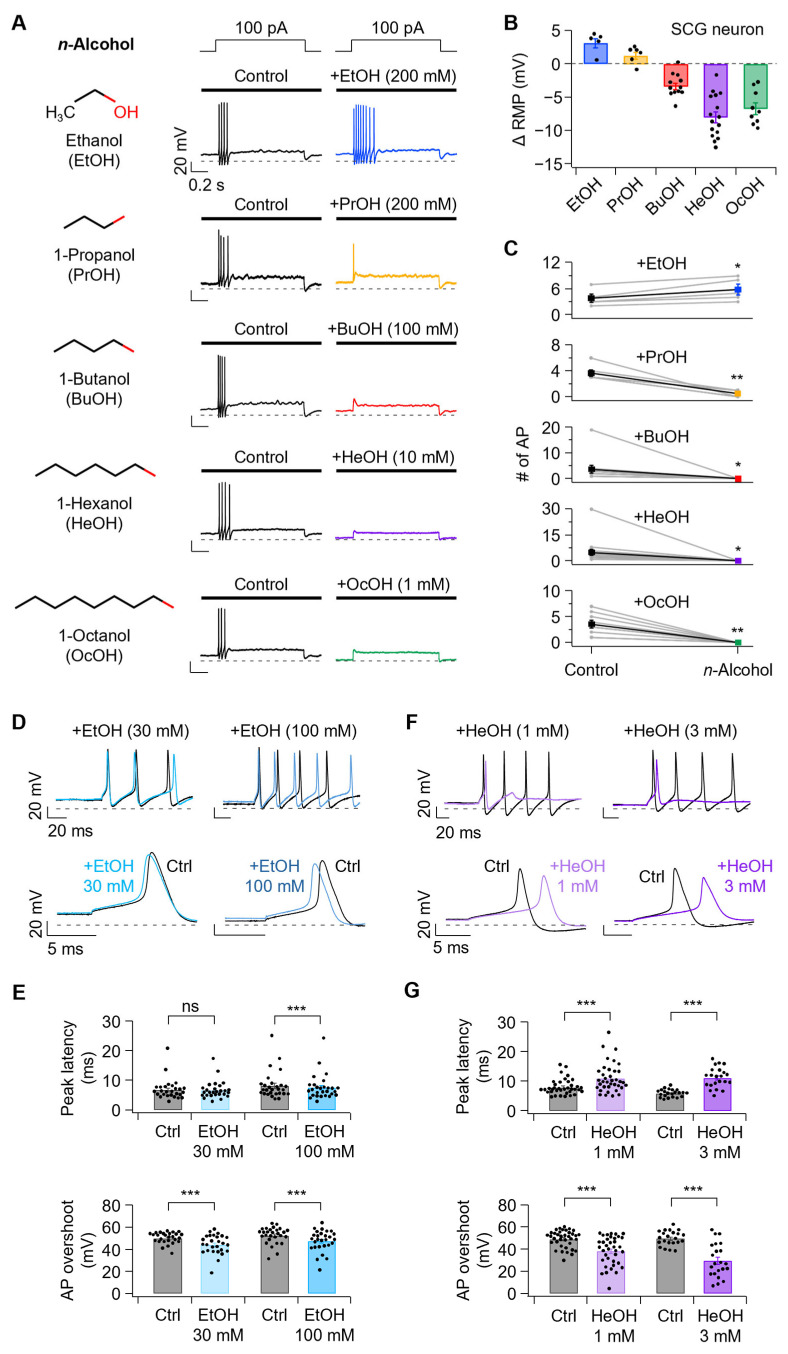
Effects of *n*-alcohols on excitability parameters of rat superior cervical ganglion (SCG) neurons. (**A**) **Left**, the chemical structure of *n*-alcohols used in this study. **Right**, representative traces of action potential (AP) evoked by a 100 pA current injection in SCG neurons before and after treatment with ethanol (EtOH, 200 mM, blue), propanol (PrOH, 200 mM, yellow), butanol (BuOH, 100 mM, red), hexanol (HeOH, 10 mM, purple) and octanol (OcOH, 1 mM, green). (**B**) Changes in resting membrane potential (ΔRMP) following *n*-alcohol application (EtOH, *n* = 5; PrOH, *n* = 6; BuOH, *n* = 12; HeOH, *n* = 16; and OcOH, *n* = 9). (**C**) Action potential frequency before and after *n*-alcohol application (EtOH, *n* = 5, *p* = 0.0217; PrOH, *n* = 6, *p* = 0.00327; BuOH, *n* = 12, *p* = 0.0256; HeOH, *n* = 16, *p* = 0.0119; and OcOH, *n* = 9, *p* = 0.00104). (**D**) Following treatment with EtOH 30 and 100 mM, the overlay traces of the initial few action potentials are shown (**top**), with the first action potential displayed on a magnified time scale (**bottom**). (**E**) Effects of EtOH (30 mM, *n* = 27; 100 mM, *n* = 28) on AP peak latency (30 mM, *p* = 0.238; 100 mM, *p* = 0.00076) and overshoot (30 mM, *p* = 6.0 × 10^−7^; 100 mM, *p* = 4.4 × 10^−9^). (**F**) The overlay traces of the initial few action potentials following treatment with HeOH 1 and 3 mM are shown (**top**), with the first action potential displayed on an enhanced time scale (**below**). (**G**) Effects of HeOH (1 mM, *n* = 37; 3 mM, *n* = 22) on AP peak latency (1 mM, *p* = 7.2 × 10^−9^; 3 mM, *p* = 5.8 × 10^−9^) and overshoot (1 mM, *p* = 3.7 × 10^−11^; 3 mM, *p* = 5.8 × 10^−9^). Statistical significance was assessed using paired Student’s *t*-test for (**C**,**E**,**G**). Statistical significance is denoted as ns, no significance; * *p* < 0.05, ** *p* < 0.01, *** *p* < 0.001.

To assess the overall effects of *n*-alcohols on neuronal excitability, we first treated various types of *n*-alcohols to rat SCG neurons. We examined resting membrane potential (RMP) and AP firing changes. Under conditions of 100 pA current injection for 1 s, 200 mM EtOH increased AP firing from 3.8 ± 1.0 to 5.8 ± 1.3, while 200 mM PrOH decreased AP firing from 3.7 ± 0.5 to 0.5 ± 0.2. Treatment with 100 mM BuOH, 10 mM HeOH, and 1 mM OcOH completely silenced AP firing from baseline values of 3.7 ± 1.4, 4.9 ± 1.7, and 3.6 ± 0.7, respectively (Figure 1A,C). Concurrent with changes in AP firing, RMP was also altered with EtOH and PrOH treatment, resulting in RMP increases of 3.1 ± 0.7 and 1.2 ± 0.5 mV, respectively, compared to baseline. In contrast, BuOH, HeOH, and OcOH decreased RMP by −3.4 ± 0.5, −8.0 ± 0.8, and −6.7 ± 0.9 mV, respectively (Figure 1B).

Based on our previous experimental results, *n*-alcohols differentially regulate Kv7 currents according to chain length, with short-chain alcohols inhibiting Kv7 currents and long-chain alcohols stimulating them [25]. Since the regulation of Kv7 currents by *n*-alcohols was not completely consistent with AP firing patterns, this suggests that *n*-alcohols may modulate the activity of various ion channels. Therefore, we sought to determine how *n*-alcohols regulate AP firing. EtOH, which increases AP firing, and HeOH, which silences AP firing, were selected as model compounds for further mechanistic investigation.

According to experimental results using tadpoles, the concentration of EtOH showing anesthetic effects was 190 mM [1], and 100 mM EtOH corresponds to approximately 0.46% blood alcohol concentration, which poses a high possibility of death [35]. Therefore, considering the physiological toxicity concerns of 200 mM EtOH, we compared AP firing at reduced concentrations of 30 mM and 100 mM. Both concentrations increased AP firing (Figure 1D and Figure S1), but 100 mM EtOH produced more robust effects across all AP parameters (Figure 1E and Figure S1). Comparison of the expanded first AP revealed that EtOH treatment affected AP initial peak and overshoot. At 100 mM, EtOH significantly accelerated the peak latency from 8.1 ± 0.9 ms to 7.5 ± 0.8 ms, while 30 mM EtOH showed no significant effect (6.7 ± 0.7 ms to 6.6 ± 0.6 ms). Both concentrations significantly reduced AP overshoot. At 30 mM, EtOH decreased AP overshoot from 50.0 ± 1.0 mV to 44.1 ± 1.7 mV, and 100 mM EtOH decreased it from 52.1 ± 1.4 mV to 47.2 ± 1.8 mV (Figure 1E). Therefore, we selected 100 mM EtOH for mechanistic studies to ensure robust suitable for dissecting the relative contributions of Na_V_ and Kv7 channels to alcohol-induced excitability changes.

Hexanol is not an endogenous compound and thus does not have a defined physiological concentration. Instead, hexanol has been widely used as a model *n*-alcohol to probe chain length–dependent effects on ion channels. Previous studies typically applied hexanol in the 1–10 mM range in neuronal and expression system experiments [36,37], which is consistent with the concentrations we studied. Since high concentrations of HeOH completely silenced AP firing (Figure 1A,C), making additional analysis difficult, we tested AP firing at reduced concentrations of 1 mM and 3 mM. Treatment with 1 and 3 mM HeOH decreased AP firing (Figure 1F). Comparison of the expanded first AP before and after HeOH treatment revealed that the time to reach the initial peak was delayed, and AP overshoot was decreased compared to baseline. HeOH treatment at 1 and 3 mM delayed AP peak latency from 7.8 ± 0.4 ms to 10.9 ± 0.8 ms and from 5.8 ± 0.3 ms to 11.0 ± 0.7 ms, respectively, and decreased overshoot from 48.7 ± 1.3 mV to 38.1 ± 2.1 mV and from 49.4 ± 1.4 mV to 29.4 ± 3.2 mV, respectively (Figure 1G).

### 3.2. Both EtOH and HeOH Suppress Sodium Currents, Confirming Previous Findings

**Figure 2:**
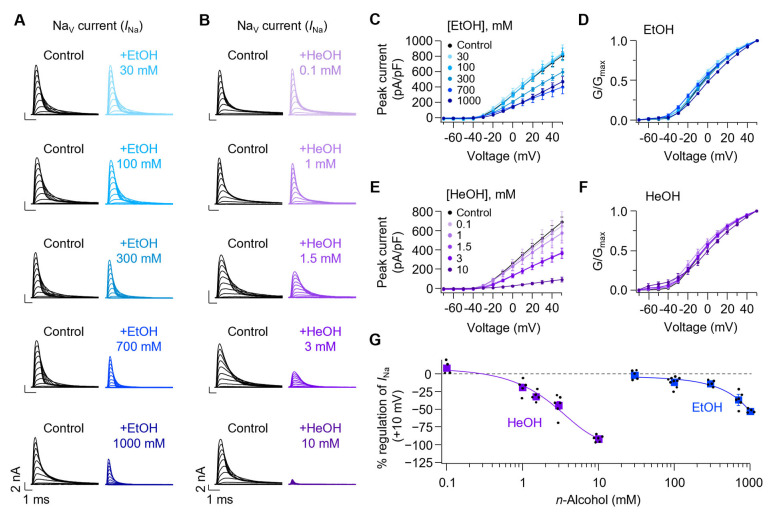
Effect of ethanol and hexanol on sodium currents in rat SCG neurons. (**A**) Representative trace of Na_V_ currents before and after application of EtOH (30–1000 mM; 100 mM ≈ 0.46% blood alcohol). Concentrations above 300 mM exceed physiological relevance and are expected to be toxic. (**B**) Representative Na_V_ current traces before and after HeOH treatment. (**C**) Current-voltage (I–V) relationships of peak Na_V_ current density before and after application of EtOH (30 mM, *n* = 5; 100 mM, *n* = 9; 300 mM, *n* = 5; 700 mM, *n* = 6; 1000 mM, *n* = 4). (**D**) Normalized conductance (G/G_max_) curve before and after EtOH treatment (30 mM, *n* = 5; 100 mM, *n* = 9; 300 mM, *n* = 5; 700 mM, *n* = 6; 1000 mM, *n* = 4). (**E**) I-V relationships of Na_V_ peak current density before and after HeOH application (0.1 mM, *n* = 4; 1 mM, *n* = 5; 1.5 mM, *n* = 5; 3 mM, *n* = 6; 10 mM, *n* = 6). (**F**) The normalized conductance (G/G_max_) was calculated under control conditions and following HeOH treatment (0.1 mM, *n* = 4; 1 mM, *n* = 5; 1.5 mM, *n* = 5; 3 mM, *n* = 6; 10 mM, *n* = 6). (**G**) Dose–response curves of EtOH and HeOH on sodium peak current amplitudes measured at + 10 mV (EtOH, *n* = 4–9; HeOH, *n* = 4–6).

Consistent with previous studies [5,6,7,8], we confirmed that *n*-alcohols modulate voltage-gated sodium channels in SCG neurons. Both EtOH (Figure 2A,E) and HeOH (Figure 2B,C) reduced peak sodium current in a concentration-dependent manner, with HeOH exerting a more potent inhibitory effect at comparable concentrations. EtOH at 30, 100, 300, 700, and 1000 mM inhibited sodium peak current at +10 mV by 1.9 ± 2.9, 11.9 ± 2.3, 14.0 ± 1.9, 36.8 ± 4.3, and 52.9 ± 1.4%, respectively. HeOH at 0.1, 1, 1.5, 3, and 10 mM inhibited sodium peak current by −8.3 ± 4.6, 19.1 ± 4.6, 32.2 ± 3.1, 44.8 ± 5.7, and 92.0 ± 1.7%, respectively (Figure 2G). Consistent with previous studies using Xenopus oocytes expressing sodium channels [8], our results confirmed that alcohols inhibit sodium channels with greater potency at longer chain lengths.

Boltzmann fitting of normalized conductance curves before and after EtOH and HeOH treatment (Figure 2D,F and Figure S2) revealed rightward shifts of 2.3 ± 0.8 and 5.0 ± 0.8 mV for 700 mM and 1000 mM EtOH, respectively, compared to baseline. Treatment with 3 mM and 10 mM HeOH also resulted in rightward shifts of 2.5 ± 0.8 and 8.2 ± 1.8 mV, respectively. These results suggest that shifts in activation properties also accompany current suppression by high concentrations of EtOH and HeOH.

### 3.3. Kv7 Currents Are Oppositely Regulated by EtOH and HeOH

**Figure 3:**
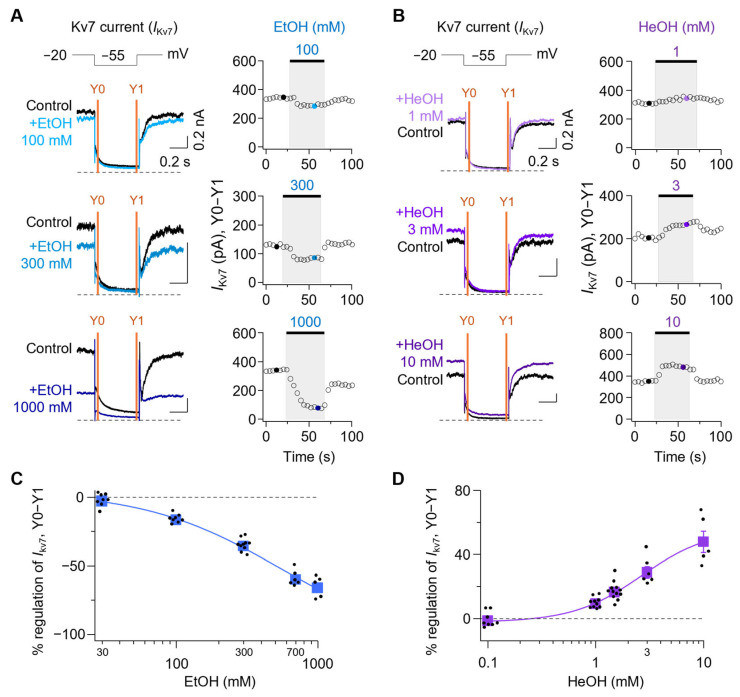
Effect of ethanol and hexanol on Kv7 channels in rat SCG neurons. (**A**) **Left**, representative traces showing Kv7 current traces during EtOH treatment. The dashed line indicates the zero-current level. **Right**, time-course analysis of *I*_Kv7_(Y0−Y1) following sequential treatments of 100, 300, and 1000 mM EtOH. (**B**) **Left**, representative traces showing Kv7 currents elicited in neurons while the cells were treated with HeOH. **Right**, time-course of Kv7 current (Y0−Y1) regulation by 1, 3, and 10 mM HeOH. The concentration−response relationship shows the inhibitory effect of EtOH (*n* = 5–10) (**C**) and the stimulatory effect of HeOH (*n* = 5–12) (**D**) on the Kv7 current amplitude in SCG neurons.

Next, we tested how M-type potassium currents, which are mainly mediated by Kv7 channels in SCG neurons, are modulated by EtOH and HeOH. The *I*_Kv7_ was calculated by subtracting the average current amplitude at 10 ms before the end of the tail current (Y1) from that at 5–10 ms after the peak of the current (Y0) and is denoted as *I*_Kv7_(Y0–Y1) (Figure 3A,B). Time-course analysis revealed that *I*_Kv7_(Y0–Y1) was inhibited by 2.8 ± 1.6%, 16.4 ± 1.1%, 35.5 ± 1.4%, 59.7 ± 2.3%, and 65.9 ± 3.5% with 30, 100, 300, 700, and 1000 mM EtOH, respectively (Figure 3C). Conversely, treatment with 0.1, 1, 1.5, 3, and 10 mM HeOH resulted in change of *I*_kv7_(Y0-Y1) by −1.3 ± 1.7%, 9.3 ± 1.0%, 16.7 ± 1.6%, 29.0 ± 3.5%, and 48.0 ± 6.8%, respectively (Figure 3D). Given the established role of Kv7 channels in regulating neuronal excitability, these results suggest that modulation of M-currents may underlie the divergent AP firing patterns induced by EtOH and HeOH.

### 3.4. Comparison of EtOH and HeOH Effects with Selective Na_V_ and Kv7 Channel Modulation

**Figure 4:**
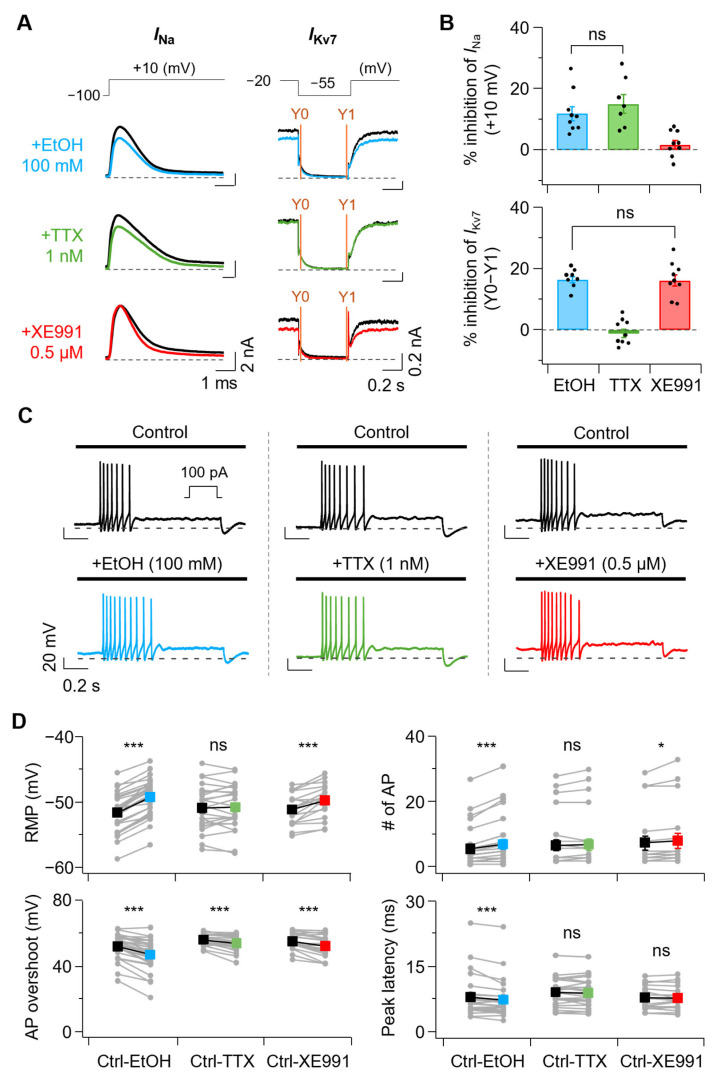
Comparative analysis of ethanol and channel modulators that mimic its effects on Na_V_ and Kv7 currents and their effects on AP properties. (**A**) **Left**, representative traces of Na_V_ currents were recorded at +10 mV under control (black) and following treatment with EtOH (100 mM, blue), TTX (1 nM, green), and XE991 (0.5 μM, red). **Right***,* representative Kv7 current traces before (black) and after treatment with EtOH, TTX, and XE991. (**B**) **Top**, summary of Na_V_ current inhibition by each treatment (EtOH, *n* = 9; TTX, *n* = 7; XE991, *n* = 9; EtOH vs. TTX, *p* = 0.595). **Bottom**, percent inhibition of Kv7 currents (Y0-Y1) following each treatment (EtOH, *n* = 8; TTX, *n* = 9; XE991, *n* = 9; EtOH vs. XE991, *p* = 0.993). (**C**) Representative AP traces before and after EtOH, TTX, and XE991 application. (**D**) Summary of changes in AP properties induced by each treatment. Parameters (**top** to **bottom**): RMP (Ctrl-EtOH, *n* = 28, *p* = 2.8 × 10^−14^; Ctrl-TTX, *n* = 24, *p* = 0.682; Ctrl-XE991, *n* = 18, *p* = 0.000133), number of AP (Ctrl-EtOH, *n* = 28, *p* = 7.5 × 10^−5^; Ctrl-TTX, *n* = 24, *p* = 0.328; Ctrl-XE991, *n* = 18, *p* = 0.0229), AP overshoot (Ctrl-EtOH, *n* = 28, *p* = 4.4 × 10^−9^; Ctrl-TTX, *n* = 24, *p* = 0.000124; Ctrl-XE991, *n* = 18, *p* = 0.000362), peak latency (Ctrl-EtOH, *n* = 28, *p* = 0.000759; Ctrl-TTX, *n* = 24, *p* = 0.417; Ctrl-XE991, *n* = 18, *p* = 0.571). Statistical significance was assessed using one-way ANOVA (**B**) with Post hoc analysis using Tukey’s HSD and paired Student’s *t*-test (**D**). ns, no significance; * *p* < 0.05, *** *p* < 0.001.

To test whether the effects of EtOH and HeOH on AP firing properties could be mimicked by selective modulation of Na_V_ or Kv7 channels, we compared their actions with known pharmacological compounds. Since 100 mM EtOH inhibited sodium and Kv7 currents by 11.9 ± 2.3% and 16.4 ± 1.1%, respectively (Figure 4A,B, blue), we determined concentrations of compounds that would modulate Na_V_ and Kv7 currents to similar extents as EtOH. Treatment with 1 nM TTX, a sodium channel blocker, reduced Na_V_ current by 15.1 ± 3.1%, showing a similar level to EtOH, but Kv7 current inhibition was minimal at −1.1 ± 1.4% (Figure 4A,B, green). In contrast, treatment with 0.5 μM XE991, a Kv7 channel blocker, showed minimal Na_V_ current inhibition at 1.7 ± 1.4% but reduced Kv7 current by 16.1 ± 1.9%, similar to EtOH levels (Figure 4A,B, red). XE991 0.5 μM treatment did not significantly change total sodium current at +10 mV from 6.27 ± 0.8 nA to 6.16 ± 0.8 nA, and no significant shift was observed in the normalized conductance curve (Figure S3E,H).

Comparison of AP firing changes before and after treatment with 100 mM EtOH, 1 nM TTX, and 0.5 μM XE991 revealed that EtOH and XE991 treatment increased AP firing. In contrast, TTX treatment showed no change in AP firing (Figure 4C). Various AP properties were examined before and after treatment with each compound (Figure 4D). RMP was calculated by averaging voltage values in the 50–52 ms range prior to 100 pA current injection. Treatment with 100 mM EtOH increased RMP from −51.6 ± 0.6 mV to −49.2 ± 0.5 mV. XE991 0.5 μM also increased RMP from −51.2 ± 0.6 mV to −49.8 ± 0.6 mV, while 1 nM TTX showed no significant increase from −50.9 ± 0.7 mV to −50.8 ± 0.7 mV. Consistent with RMP changes, AP numbers changed accordingly: EtOH treatment increased from 5.8 ± 1.2 to 7.2 ± 1.4, XE991 treatment increased from 7.6 ± 2.1 to 8.2 ± 2.3, while TTX treatment showed no significant increase from 6.8 ± 1.7 to 7.0 ± 1.8.

AP overshoot, calculated as the amplitude from 0 mV to peak, decreased with EtOH from 52.1 ± 1.4 mV to 47.2 ± 1.8 mV, with TTX and XE991 causing decreases from 56.1 ± 0.7 mV to 54.1 ± 1.0 mV and from 55.2 ± 1.2 mV to 52.3 ± 1.6 mV, respectively. Peak latency (time to reach first AP peak) was decreased by EtOH from 8.1 ± 0.9 ms to 7.5 ± 0.8 ms, while TTX and XE991 showed no significant changes from 9.1 ± 0.6 ms to 9.0 ± 0.7 ms and from 7.9 ± 0.6 ms to 7.9 ± 0.6 ms, respectively.

**Figure 5:**
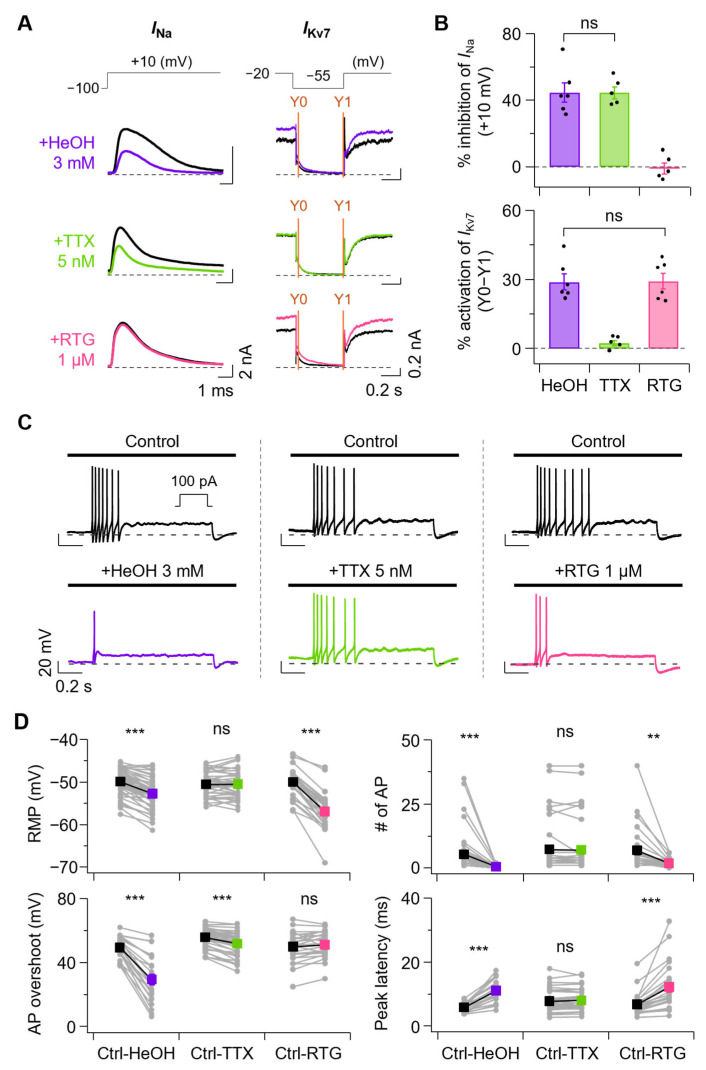
Comparative analysis of hexanol and channel modulators mimicking its effects on Na_V_ and Kv7 currents and their influence on AP properties. (**A**) **Left**, representative traces of Na_V_ currents recorded at +10 mV under control (black) and following treatment with HeOH (3 mM, purple), TTX (5 nM, yellow-green), and RTG (1 μM, magenta) and **Right**, Representative traces of Kv7 current before and after treatment with HeOH, TTX, and RTG at the indicated concentrations. (**B**) **Top**, percent inhibition of peak Na_V_ currents by each treatment (HeOH, *n* = 6; TTX, *n* = 5; RTG, *n* = 5; HeOH vs. TTX, *p* > 0.999). **Bottom**, percent activation of Kv7 currents (Y0–Y1) after each treatment (HeOH, *n* = 6; TTX, *n* = 5; RTG, *n* = 6; HeOH vs. RTG, *p* = 0.999). (**C**) Representative traces of APs were recorded before and after the application of HeOH, TTX, and RTG. (**D**) Summary of treatment-induced changes in AP properties. Parameters are shown from top to bottom: RMP (Ctrl-HeOH, *n* = 48, *p* = 2.3 × 10^−15^; Ctrl-TTX, *n* = 38, *p* = 0.804; Ctrl-RTG, *n* = 27, *p* = 4.9 × 10^−14^), number of AP (Ctrl-HeOH, *n* = 48, *p* = 2.2 × 10^−5^; Ctrl-TTX, *n* = 38, *p* = 0.404; Ctrl-RTG, *n* = 27, *p* = 0.00132), AP overshoot (Ctrl-HeOH, *n* = 22, *p* = 5.8 × 10^−9^; Ctrl-TTX, *n* = 38, *p* = 5.8 × 10^−11^; Ctrl-RTG, *n* = 25, *p* = 0.102), peak latency (Ctrl-HeOH, *n* = 22, *p* = 5.8 × 10^−9^; Ctrl-TTX, *n* = 38, *p* = 0.183; Ctrl-RTG, *n* = 25, *p* = 1.3 × 10^−5^). Statistical significance was assessed using one-way ANOVA (**B**) with Post hoc analysis using Tukey’s HSD and paired Student’s *t*-test (**D**). ns, no significance; ** *p* < 0.01, *** *p* < 0.001.

Next, since 3 mM HeOH inhibited sodium current by 44.8 ± 5.7% and activated Kv7 current by 29.0 ± 3.5% (Figure 5A,B, purple), we determined concentrations of compounds that would modulate sodium and Kv7 currents to similar extents as HeOH. Treatment with 5 nM TTX reduced Na_V_ current by 44.7 ± 3.5%, showing a similar level to HeOH, but Kv7 current change by TTX 5 nM was minimal at −2.3 ± 1.2% (Figure 5A,B, yellow-green). In contrast, treatment with 1 μM retigabine (RTG), a well-known Kv7 activator except for Kv7.1 [38,39,40], showed minimal Na_V_ current change at −0.9 ± 3.3% but activated Kv7 current by 29.2 ± 3.4%, similar to HeOH levels (Figure 5A,B, magenta). RTG 1 μM treatment did not significantly change the total sodium current at +10 mV from 6.14 ± 0.9 nA to 6.19 ± 0.9 nA, and no significant shift was observed in the normalized conductance curve (Figure S3A–D).

Comparison of AP firing changes before and after treatment with 3 mM HeOH, 5 nM TTX, and 1 μM RTG revealed that HeOH and RTG treatment decreased AP firing. In comparison, TTX treatment showed no change in AP firing (Figure 5C). Various AP properties were examined before and after treatment with each compound (Figure 5D). Treatment with 3 mM HeOH decreased RMP from −49.9 ± 0.4 mV to −52.8 ± 0.5 mV. RTG 1 μM also decreased RMP from −50.0 ± 0.7 mV to −56.9 ± 0.8 mV, while 5 nM TTX showed no significant change from −50.6 ± 0.5 mV to −50.5 ± 0.5 mV. Consistent with RMP changes, AP numbers changed accordingly: HeOH treatment decreased from 5.3 ± 1.1 to 0.5 ± 0.1, RTG treatment decreased from 6.9 ± 1.7 to 1.8 ± 0.3, while TTX treatment showed no significant change from 7.3 ± 1.6 to 7.0 ± 1.6.

We compared AP overshoot changes before and after treatment. HeOH decreased overshoot from 49.4 ± 1.4 mV to 29.4 ± 3.2 mV, TTX treatment decreased from 55.8 ± 1.0 mV to 51.9 ± 1.2 mV, while RTG induced no significant change from 49.9 ± 1.9 mV to 51.2 ± 1.6 mV. HeOH and RTG significantly increased peak latency from 5.8 ± 0.3 ms to 11.0 ± 0.7 ms and from 6.8 ± 0.7 ms to 12.2 ± 1.4 ms, respectively, while TTX treatment showed no significant change from 7.8 ± 0.5 ms to 8.0 ± 0.5 ms.

### 3.5. A Combination of Na_V_ and Kv7 Modulators Could Mimic EtOH and HeOH

**Figure 6:**
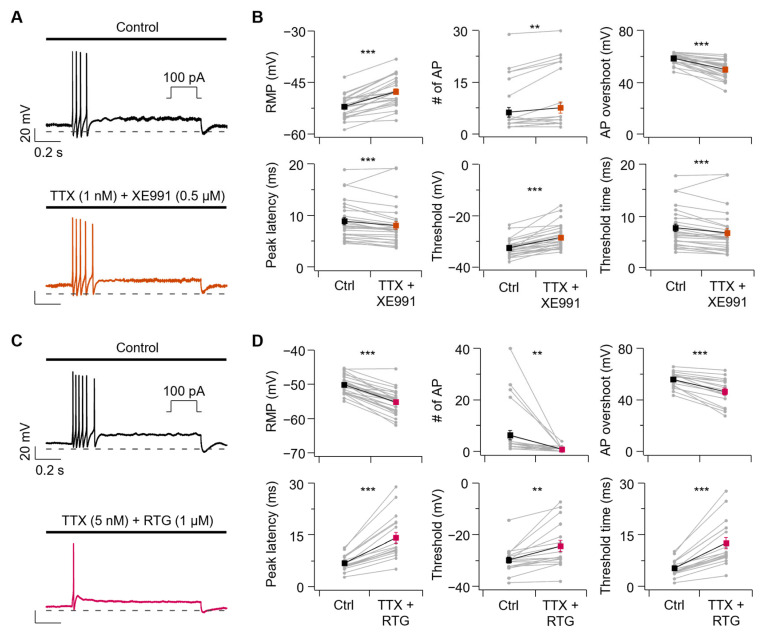
Combined modulation of Na_V_ and Kv7 channels and their effects on action potential waveform and parameters. (**A**) Representative AP traces before and after co-application of TTX (1 nM) and XE991 (0.5 µM). (**B**) Summary of changes in AP properties before and after combined treatment. Parameters shown from top to bottom: RMP (Ctrl-TTX + XE991, *n* = 27, *p* = 5.6 × 10^−10^), number of AP (Ctrl-TTX + XE991, *n* = 27, *p* = 0.00362), AP overshoot (Ctrl-TTX + XE991, *n* = 27, *p* = 1.3 × 10^−8^), peak latency (Ctrl-TTX + XE991, *n* = 27, *p* = 0.000978), threshold (Ctrl-TTX + XE991, *n* = 27, *p* = 3.9 × 10^−7^), and threshold time (Ctrl-TTX + XE991, *n* = 27, *p* = 0.000170). (**C**) Representative AP traces before and after co-application of TTX (5 nM) and RTG (1 µM). (**D**) Summary of changes in AP properties before and after treatment. Parameters shown from top to bottom: RMP (Ctrl-TTX + RTG, *n* = 27, *p* = 1.3 × 10^−12^), number of AP (Ctrl-TTX + RTG, *n* = 27, *p* = 0.00549), AP overshoot (Ctrl-TTX + RTG, *n* = 17, *p* = 6.1 × 10^−6^), peak latency (Ctrl-TTX + RTG, *n* = 17, *p* = 4.8 × 10^−6^), threshold (Ctrl-TTX + RTG, *n* = 17, *p* = 0.00150), and threshold time (Ctrl-TTX + RTG, *n* = 17, *p* = 5.4 × 10^−6^). Statistical significance was assessed using paired Student’s *t*-test. ** *p* < 0.01, *** *p* < 0.001.

To further dissect the relative contributions of Na_V_ and Kv7 channel modulation, we tested whether dual manipulation of these channels could mimic alcohol effects. Co-application of TTX 1 nM with XE991 0.5 μM closely mimicked the effects of EtOH on AP firing properties (Figure 6A,B). RMP increased from −52.0 ± 0.6 mV to −47.6 ± 0.8 mV, and the number of APs increased from 6.2 ± 1.4 to 7.6 ± 1.6 with TTX + XE991 treatment. AP overshoot decreased from 58.6 ± 0.7 mV to 49.9 ± 1.4 mV, and peak latency was accelerated from 8.9 ± 0.7 ms to 8.1 ± 0.8 ms. Finally, the threshold increased from −32.5 ± 0.6 mV to −28.5 ± 0.9 mV, and threshold time was accelerated from 7.6 ± 0.7 ms to 6.6 ± 0.8 ms.

The effects of HeOH on AP firing properties were closely mimicked by co-applicating TTX 5 nM with RTG 1 μM (Figure 6C,D). RMP decreased from −50.1 ± 0.5 mV to −55.2 ± 0.7 mV, and the number of APs decreased from 6.3 ± 1.9 to 0.8 ± 0.2 with TTX + RTG treatment. AP overshoot decreased from 55.8 ± 1.5 mV to 46.4 ± 2.6 mV, and peak latency was delayed from 6.8 ± 0.5 ms to 14.1 ± 1.6 ms. Finally, the threshold increased from −29.7 ± 1.2 mV to −24.4 ± 2.1 mV, and threshold time was delayed from 5.4 ± 0.6 ms to 12.6 ± 1.6 ms.

### 3.6. Sodium Channel Inhibition Alone Has a Limited Impact on AP Firing in SCG Neurons

To assess how sodium current inhibition contributes to the difference in excitability changes induced by *n*-alcohols, we examined the extent to which sodium current inhibition modulates AP firing in SCG neurons. This approach allowed us to determine whether sodium channel inhibition alone could account for the changes in excitability observed with EtOH and HeOH applications.

**Figure 7:**
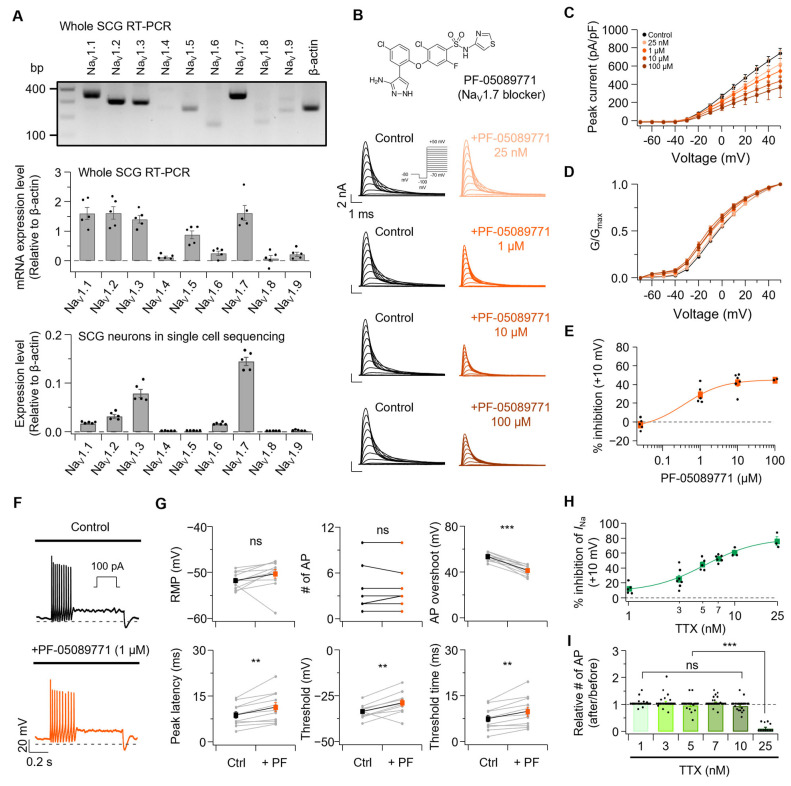
Effects of Na_V_1.7 blocker on sodium currents and AP parameters in rat SCG neurons. (**A**) Sodium channel subtype expression in neonatal rat SCG (**top**, RT-PCR) and adult mouse SCG (**bottom**, scRNA-seq), relative to ß-actin. (**B**) **Top**, structure of the Nav1.7 blocker PF-05089771. **Bottom**, representative Na_V_ current traces before and after PF-05089771 treatment. (**C**) Peak current density at +10 mV before and after PF-05089771 treatment (25 nM, *n* = 5; 1 µM, *n* = 5; 10 µM, *n* = 6; 100 µM, *n* = 2). (**D**) Normalized conductance curve of Na_V_ peak current before and after treatment with PF-05089771. (**E**) Percent inhibition of Na_V_ peak current at +10 mV after PF-05089771 treatment (*n* = 2–6). (**F**) Representative AP traces before and after treatment with PF-05089771 (1 µM). (**G**) Summary of AP properties changes before and after PF-05089771 application. Parameters shown from top to bottom: RMP (Ctrl-1 µM PF-05089771, *n* = 11, *p* = 0.128), number of AP (Ctrl-1 µM PF-05089771, *n* = 11, *p* = 0.588), AP overshoot (Ctrl-1 µM PF-05089771, *n* = 11, *p* = 3.4 × 10^−6^), peak latency (Ctrl-1 µM PF-05089771, *n* = 11, *p* = 0.00134), threshold (Ctrl-1 µM PF-05089771, *n* = 11, *p* = 0.00300), and threshold time (Ctrl-1 µM PF-05089771, *n* = 11, *p* = 0.00176). (**H**) Percent inhibition of Na_V_ peak current at +10 mV after TTX treatment (*n* = 3–7). (**I**) The ratio of AP number after treatment with TTX 1 nM (*n* = 24), 3 nM (*n* = 25), 5 nM (*n* = 21), 7 nM (*n* = 26), 10 nM (*n* = 22) and 25 nM (*n* = 11). Statistical significance was assessed using paired Student’s *t*-test for (**G**) and one-way ANOVA (**I**), which revealed a significant difference among the groups. Post hoc analysis using Tukey’s HSD test showed that the TTX 25 nM exhibited a significantly higher value than all other concentrations (1 nM, 3 nM, 5 nM, 7 nM, and 10 nM; all *p* < 0.0001). No significant differences were observed among the 1–10 nM groups (all *p* > 0.2). ns, no significance; ** *p* < 0.01, *** *p* < 0.001.

Rat SCG neurons endogenously express various sodium channel subtypes. We examined sodium channel subtypes in P5–P7 rat SCGs by comparing relative mRNA expression levels. When beta-actin expression was normalized to 1, the relative expression of Na_V_ subtypes Na_V_1.1 through Na_V_1.9 was 1.60 ± 0.21, 1.61 ± 0.21, 1.40 ± 0.13, 0.11 ± 0.03, 0.87 ± 0.14, 0.25 ± 0.07, 1.61 ± 0.25, 0.07 ± 0.11, and 0.21 ± 0.07, respectively (Figure 7A, Top). To examine the expression of Na_V_1.1–Na_V_1.9 genes in neurons, we reanalyzed single-cell RNA sequencing data from adult mouse SCGs [28]. Na_V_1.1–Na_V_1.9 genes were primarily expressed in sympathetic neurons within the SCG (Figure S4). Notably, pseudo-bulk single-cell RNA sequencing analysis revealed that Na_V_1.7 was the most abundantly expressed gene among Na_V_1.1–Na_V_1.9 in sympathetic neurons (Figure 7A, bottom; expression levels of Na_V_1.1 through Na_V_1.9 relative to beta-actin: 0.0172 ± 0.0017, 0.0315 ± 0.0079, 0.0789 ± 0.0192, 0.0005 ± 0.0004, 0.0011 ± 0.0003, 0.0152 ± 0.0024, 0.1450 ± 0.0188, 0.0005 ± 0.0002, 0.0016 ± 0.0011). Additionally, since Na_V_1.7 has been reported as the major sodium channel expressed in SCG neurons [41,42], we examined changes in total sodium current (Figure 7B–E) and AP firing properties (Figure 7F,G) before and after treatment with PF-05089771, a selective Na_V_1.7 blocker. Total sodium currents at +10 mV in SCG neurons were changed by −2.8 ± 2.6%, 29.0 ± 4.3%, 42.0 ± 4.0%, and 44.7 ± 0.5% with PF-05089771 25 nM, 1 μM, 10 μM, and 100 μM, respectively. Boltzmann fitting of normalized conductance curves before and after PF-05089771 treatment (Figure S5) revealed a significant V_1/2_ shift of −3.6 ± 0.2 mV only with 100 μM PF-05089771.

PF-05089771 (1 μM) treatment showed no significant change in RMP from −51.7 ± 0.5 mV to −50.3 ± 1.0 mV, and the number of APs remained unchanged from 3.5 ± 0.8 to 3.5 ± 0.8. AP overshoot decreased from 53.6 ± 1.0 mV to 41.5 ± 1.3 mV, and peak latency was delayed from 8.8 ± 1.1 ms to 11.4 ± 1.5 ms. Finally, the threshold increased from −33.5 ± 1.1 mV to −28.8 ± 1.8 mV, and threshold time was delayed from 7.5 ± 1.1 ms to 9.8 ± 1.5 ms. To confirm that PF-05089771, a selective Na_V_1.7 blocker, does not modulate Kv7 currents, we examined Kv7 current changes before and after treatment of PF-05089771 (Figure S6). Kv7 current amplitude in SCG neurons was changed by −3.9 ± 1.7%, 4.8 ± 1.8%, 1.9 ± 1.3%, and 4.3 ± 2.3% with PF-05089771 25 nM, 1 μM, 10 μM, and 100 μM, respectively. No significant differences between concentrations were observed.

Next, we tested sodium current changes and AP firing changes before and after treatment with TTX at 1, 3, 5, 7, 10, and 25 nM, a sodium channel blocker that can inhibit various Na_V_ subtypes. Total sodium currents at +10 mV were inhibited by 12.2 ± 3.1%, 25.5 ± 4.9%, 44.7 ± 3.5%, 52.7 ± 1.9%, 60.5 ± 3.5%, and 75.9 ± 5.9% with TTX 1, 3, 5, 7, 10, and 25 nM, respectively (Figure 7H and Figure S7). The ratios of AP number changes before and after treatment with TTX 1, 3, 5, 7, 10 and 25 nM (Figure 7I and Figure S8) were 1.03 ± 0.03, 1.07 ± 0.05, 0.97 ± 0.05, 1.06 ± 0.03, 0.94 ± 0.04, and 0.11 ± 0.05, respectively.

Importantly, substantial suppression of sodium current (up to 60.5% with 10 nM TTX) failed to alter the AP firing, with significant reduction only occurring at 25 nM TTX (75.9% inhibition). These null findings provide evidence that moderate inhibition of sodium channels is insufficient to explain the excitability changes observed with *n*-alcohol treatments, thereby supporting an alternative mechanism in which modulation of Kv7 channels drives the observed effects.

## 4. Discussion

**Figure 8:**
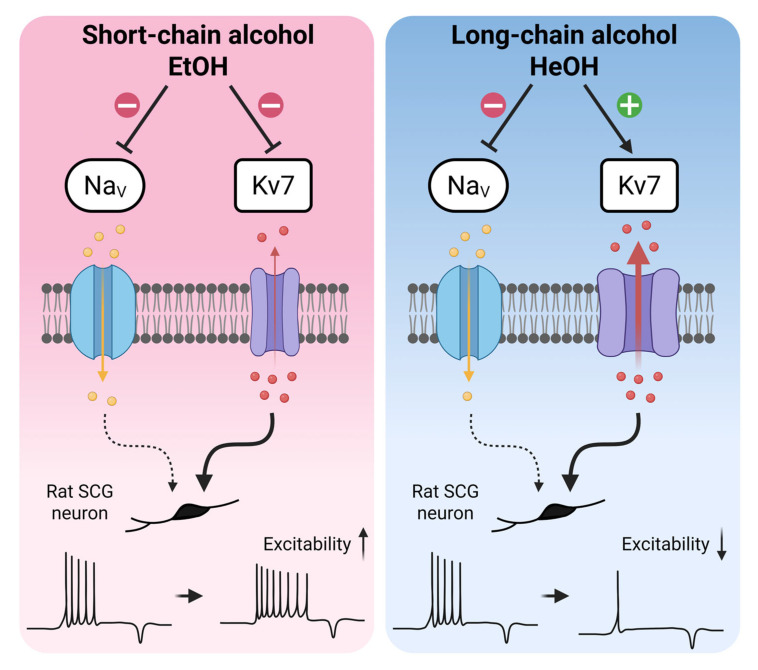
A summary diagram of the differential effects of EtOH and HeOH on the AP firing highlights the contribution of Kv7 channel modulation. EtOH inhibits Na_V_ and Kv7 channels and increases neuronal firing, whereas HeOH inhibits Na_V_ channels and activates Kv7 channels, resulting in suppressed firing in rat SCG neurons. Upward arrow indicates increased excitability; downward arrow indicates suppressed excitability. Created in BioRender. Jeong, D. (2025) https://BioRender.com/0i2s6k7 (accessed on 29 October 2025).

While sodium channel inhibition by *n*-alcohol is well-established [5,6,7,8], our findings reveal an unexpected relationship between sodium channel inhibition and neuronal excitability changes in neonatal rat SCG neurons. Both EtOH and HeOH inhibited sodium channels in a concentration-dependent manner, yet they exhibited distinct effects on AP firing. This observation differs from research in dorsal root ganglion (DRG) neurons, where ~10% sodium current inhibition typically reduces AP propagation and decreases neuronal firing [43,44]. However, because these studies were conducted in mature sensory neurons that differ from neonatal SCG neurons in ion channel composition and physiological properties, such comparisons should be considered indirect and speculative. To address this discrepancy, we conducted additional experiments using TTX at concentrations that induced sodium current suppression comparable to those observed with EtOH and HeOH treatments. Notably, substantial sodium current inhibition (up to 60.5% with 10 nM TTX) did not alter AP firing frequency, with a significant reduction only occurring at 25 nM TTX (75.9% inhibition). Similarly, PF-05089771 (1 µM), which selectively targets Na_V_1.7 channels—the predominant sodium channel subtype in SCG neurons [41,42]—achieved approximately 29% total sodium current inhibition without changing firing frequency. These findings suggest that moderate sodium current inhibition does not directly reduce excitability in neonatal rat SCG neurons. Therefore, while Na_V_ channel inhibition may be necessary, it is insufficient on its own to account for *n*-alcohol-induced changes in excitability. Additionally, *n*-alcohols differ from TTX in their selectivity profile for sodium channels. While TTX at nanomolar concentrations selectively blocks TTX-sensitive channels, *n*-alcohols can inhibit both TTX-sensitive and TTX-resistant sodium channel subtypes [7,8,45]. Our results suggest that Kv7 channel modulation is an important contributor to *n*-alcohol-induced changes in neuronal excitability. The neuronal M current, mediated by Kv7 channels, is a well-established brake on neuronal excitability [18,46,47]. EtOH inhibits Kv7 current, thereby removing this excitability brake and leading to depolarized RMP and increased AP frequency. Conversely, HeOH activates the Kv7 current, leading to RMP hyperpolarization and AP suppression. Pharmacological experiments using selective Kv7 modulators strongly support this model. Treatment with XE991 produced a similar level to inhibition of Kv7 currents as EtOH (16.1% vs. 16.4%), resulting in increased RMP and AP firing that mirrored the effects of EtOH. Similarly, RTG achieved comparable activation of Kv7 currents as HeOH (29.2% vs. 29.0%), leading to decreased RMP and AP firing. However, Kv7 modulation alone did not fully recapitulate all AP properties altered by the alcohols. To summarize these findings, we propose a schematic model illustrating the differential contributions of sodium and Kv7 currents to excitability changes induced by EtOH and HeOH (Figure 8). While both EtOH and HeOH suppress sodium currents, Kv7 modulation—suppression by EtOH and enhancement by HeOH—correlate with their respective effects on AP firing. These results indicate that Kv7 channels contribute significantly to *n*-alcohol-induced changes in neonatal rat SCG neuronal excitability and may represent important molecular targets for understanding the effects of alcohol on autonomic function.

AP characteristics provide additional mechanistic insights into neuronal excitability. The AP overshoot is known to depend on sodium channels [48]. Treatment with EtOH reduced AP overshoot to a degree comparable to the combined effects of TTX and XE991. Notably, XE991 alone also reduced AP overshoot, despite not significantly inhibiting sodium channels. This reduction likely results from depolarized RMP by XE991, which increases sodium channel inactivation and thereby reduces the number of available sodium channels, consequently decreasing AP overshoot while still allowing firing. Rapid sodium entry during the fast upstroke and rapid potassium efflux influence AP peak latency, promoting fast repolarization and preparing for the initiation of subsequent AP [49,50]. TTX at concentrations of 1 and 5 nM showed no significant changes in peak latency, indicating that sodium current inhibition at these tested concentrations did not affect this parameter. Additionally, XE991 did not produce significant changes, suggesting that the inhibition of Kv7 currents alone cannot account for the EtOH-induced change in peak latency. Thus, it is possible that EtOH influences peak latency through the modulation of additional channels beyond Na_V_ and Kv7 channels.

Analysis of threshold-RMP relationships revealed the sensitive nature of excitability control. EtOH treatment produced threshold-RMP delta values of approximately −0.01 mV, while XE991 resulted in approximately −0.4 mV. These findings indicate that even minimal reductions in the RMP-threshold gap can significantly increase firing frequency. Conversely, HeOH and RTG increased threshold-RMP delta values by approximately 8.4 mV and 6.4 mV, respectively, consistent with decreased firing due to heightened activation barriers. TTX treatments at concentrations of 1, 3, 5, 7, 10, and 25 nM yielded threshold-RMP delta values of 0.3, 0.5, 2.0, 0.2, 1.7, and 7.0 mV, respectively. This confirms that neuronal excitability suppression requires a larger gap than excitability enhancement. These results demonstrate that the regulation of Kv7 current more sensitively controls neuronal firing than sodium current inhibition in neonatal rat SCG neurons.

Our data demonstrate that *n*-alcohols exert their effects through concurrent but functionally distinct modulation of sodium and potassium channels. The chain length of *n*-alcohols influences the magnitude and direction of Kv7 current modulation. Short-chain alcohols, such as EtOH, inhibit Kv7 currents, whereas long-chain alcohols, like HeOH, stimulate them. This bidirectional modulation accounts for the biphasic effects of *n*-alcohols on neuronal excitability. We validated this dual-target model through combinations of selective channel modulators: co-application of TTX and XE991 effectively mimicked the effects of EtOH, while TTX combined with RTG recapitulated the effects of HeOH. Importantly, neither sodium channel blockade nor Kv7 modulation alone fully reproduced the spectrum of changes induced by alcohol treatment.

SCG neurons serve as a valuable model for studying ion channel regulation in the peripheral sympathetic nervous system [51,52,53]. However, several considerations are necessary when interpreting our results. First, we conducted experiments using neonatal (P5–P7) rat SCG neurons, which may differ from adult neurons in ion channel expression and excitability properties. The use of neonatal neurons is a common approach in electrophysiological studies of SCG because these cells can be reliably dissociated and maintained in culture, allowing stable whole-cell recordings. This methodological choice was based on technical feasibility rather than an attempt to model fetal ethanol exposure. Notably, postnatal development is accompanied by changes in K^+^ current expression [54] and receptor expression levels, such as ryanodine receptor 3, which vary with age in rat SCG [55]. Although our data suggests some conservation of Na_V_ channel expression between neonatal rat and adult mouse SCG, extrapolation of these findings to adult or sensory neuronal physiology remains speculative. Future studies using adult rat SCG neurons will be important to confirm whether the present findings extend to mature sympathetic neurons. In addition, because mixed-sex litters were used, potential sex-dependent difference could not be assessed and should be addressed in future work. Second, our results may be specific to SCG neurons. Kv7 current magnitude has been shown to strongly correlate with firing patterns and RMP in SCG neurons [22]. The role of the Kv7 channel as an important contributor of excitability in our study may reflect unique properties of this neuronal population. Consistent with this notion, ketamine exhibits cell type-specific effects on Kv7.2 channels, inhibiting or activating them depending on the brain region and cell type [56]. This suggests that the same compound can produce different responses depending on cell types. Future studies examining the effects of *n*-alcohol across diverse neuronal populations will be necessary to establish the generalizability of our findings. Third, ethanol is known to modulate diverse molecular targets in neurons. In addition to voltage-gated ion channels, ethanol affects ligand-gated ion channels including NMDA [9,10], GABA_A_ [11,12], and glycine receptors [13,14]. Ethanol also modulates multiple K^+^ channel subtypes beyond Kv7, including Shaw2 Kv [57], G protein-coupled inwardly rectifying potassium (GIRK) [58], BK [59], SK [60], and IK_Ca_ channels [61]. While SCG neurons may express several K^+^ channels at varying densities, the physiological response under native conditions may represent the net effect of multiple ion channel modulations. Therefore, our conclusions are specifically relevant to isolated ion channel function in neonatal rat SCG neurons and should not be directly extrapolated to mature neuronal populations or systemic alcohol effects.

High concentrations of alcohol could potentially exert non-specific effects on ion conductance and membrane properties. Ethanol has been reported to increase membrane fluidity, reduce structural stability, and affect protein structure and function [62,63,64]. Although 100 mM EtOH exceeds physiological levels, several lines of evidence suggest that non-specific effects were minimal under our experimental conditions. First, we applied alcohol for short durations. Second, recording stability parameters were consistent with specific channel modulation rather than nonspecific membrane disruption. Series resistance (R_S_) remained stable throughout EtOH and HeOH application, indicating that seal quality was maintained (Figure S9). In contrast, input resistance (R_in_) changed bidirectionally, consistent with their opposing modulatory actions on Kv7 channels rather than nonspecific membrane perturbation. Third, previous studies showed that even 400 mM ethanol specifically inhibits Kv7.2/7.3 channels without disrupting GPCR signaling [22]. And our data demonstrates that following 10 mM hexanol treatment, GPCR modulation of Kv7.2/7.3 channels remained intact (Figure S10). Consistent with previous results showing that the inhibitory effect of EtOH on Kv7.2/7.3 channels is attenuated by elevated PI(4,5)P_2_ levels [22], we found that overexpression of PIPKIγ similarly diminished the stimulatory effect of HeOH on Kv7.2/7.3 currents (Figure S10). This parallel antagonism by elevated PI(4,5)P_2_ suggests that both EtOH and HeOH modulate Kv7 channels through specific interactions with the PI(4,5)P_2_-channel regulatory pathway rather than through alterations in membrane fluidity or protein structural changes. These findings suggest that non-specific effects did not significantly contribute to our results. Kv7 channels play an important role in regulating neuronal excitability. Therefore, alcohol-induced modulation of Kv7 currents in SCG neurons may have broader implications for systemic responses, potentially contributing to differences in autonomic tone, sedation, or anesthetic sensitivity observed with various alcohols.

Our findings demonstrate that the direction of Kv7 channel modulation is a critical contributor of excitability changes in neonatal rat SCG neurons. Although *n*-alcohols uniformly inhibit sodium currents regardless of chain length, their effects on Kv7 channels vary depending on chain length, accounting for the contrasting impacts on neuronal excitability. These insights highlight Kv7 channels as a potential target for controlling neuronal excitability.

## 5. Conclusions

In summary, our findings identify Kv7 channels as an important contributor to alcohol-induced changes in neuronal excitability. Given the widespread expression of Kv7 channels throughout the nervous system and their fundamental role in controlling neuronal excitability, these mechanisms may contribute to alcohol action in other neuronal populations and could influence autonomic function, pain modulation, and potentially behavioral responses to alcohol. Future studies examining Kv7 channel modulation in adult neurons and different neuronal subtypes will be important for determining the generalizability and systemic relevance of these findings.

## Data Availability

All the data in the present study are available from the corresponding author upon reasonable request.

## Supplementary Materials

The following supporting information can be downloaded at: https://www.mdpi.com/article/10.3390/cells14211723/s1, Supplementary figures and supplementary methods are provided in the Supplementary information (Methods and Figures S1–S10). Figure S1 shows the effects of EtOH 30 mM on rat SCG neurons. Figure S2 shows the effects of EtOH and HeOH on the Boltzmann-fitted activation curve. Figure S3 shows the insensitivity of sodium channels in SCG neurons to RTG and XE991. Figure S4 shows the expression levels of Nav1.1–1.9 genes across the different cell types of the SCG. Figure S5 shows the effects of PF-05089771 on the Boltzmann fitted activation curve. Figure S6 shows the effects of PF-05089771 on Kv7 channels in SCG neurons. Figure S7 shows the dose-dependent inhibition of sodium currents by TTX. Figure S8 shows the representative AP traces with or without TTX. Figure S9 shows recording stability and bidirectional modulation during treatment with EtOH and HeOH. Figure S10 shows PIP_2_-dependent regulation of HeOH effects on Kv7.2/7.3 channels and GPCR signaling, [28,65,66].

## Abbreviations

The following abbreviations are used in this manuscript:

AP	Action potential
BuOH	Butanol
EtOH	Ethanol
HeOH	Hexanol
Kv7	Voltage-gated potassium channel subfamily 7 (KCNQ channels)
Na_V_	Voltage-gated sodium channel
OcOH	Octanol
PI(4,5)P_2_	Phosphatidylinositol 4,5-bisphosphate
PIPKIγ	Phosphatidylinositol-4-phosphate 5-kinase type Iγ
PrOH	Propanol
RMP	Resting membrane potential
RTG	Retigabine
SCG	Superior cervical ganglion
