# Supplementary material for: Kv7 Channels as an Important Contributor to Alcohol-Induced Modulation of Neuronal Excitability in Neonatal Rat Superior Cervical Ganglion
Source: Cells. 2025 Nov 1;14(21):1723. doi: 10.3390/cells14211723 (PMC12609967; doi:10.3390/cells14211723)
Supplement: Supplementary file 1 [file cells-14-01723-s001.zip › cells-3939828-supplementary.pdf]

Supplementary Information

Kv7 Channels as an Important Contributor to Alcohol-induced Modulation of Neuronal Excitability in Neonatal Rat Superior Cervical Ganglion

Da-Jeong Jeong, Jin-Nyeong Woo, Tery Yun, Myungin Baek, Byung-Chang Suh

Include:

Supplementary Methods

Supplementary Figures S1-10

Supplementary Methods

Single-cell RNA sequencing data analysis

Single-cell RNA Sequencing Data Processing

Publicly available Drop-Seq data from the SCG of adult mice [28] were reanalyzed. Five biological replicates (SCG_rep1 through SCG_rep5) were included in the analysis. A genome index was built with STAR v2.7.11a [65] using the Mus_musculus.GRCm39.dna.primary_assembly.fa reference and the corresponding Mus_musculus.GRCm39.113.gtf annotation (Ensembl release 113).

Alignment and quantification were performed using the STARsolo [66] module with parameters optimized for Drop-seq data: cell barcodes (12 nt) extracted from positions 1–12 (--soloCBstart 1; --soloCBlen 12) and UMI sequences (8 nt) from positions 13–20 (--soloUMIstart 13; --soloUMIlen 8) on Read 1, adapter clipping via CellRanger4 mode (--clipAdapterType CellRanger4), exact UMI deduplication (--soloUMIdedup Exact), unstranded alignment (--soloStrand Unstranded), restriction of multimapping to one locus (--outFilterMultimapNmax 1), and a minimum alignment score of 30 (--outFilterScoreMin 30). Gene-level counts were generated (--quantMode GeneCounts), and output files, including BAM alignments and cell-by-gene count matrices, were formatted in a Cell Ranger-compatible structure.

Quality Control of Single-cell RNA-seq Data

Gene-by-cell count matrices produced by STARsolo were imported into R (v4.3.3) using the read10xCounts() function from the DropletUtils package (version 1.22.0), and converted into a SingleCellExperiment object (version 1.24.0). Feature names were uniquified based on Ensembl IDs and gene symbols via uniquifyFeatureNames(), and cell barcodes were assigned as column names.

Barcode rank plots were generated using DropletUtils::barcodeRanks() to assess UMI count distributions and distinguish true cell-containing barcodes from background. The emptyDrops() algorithm was applied with a fixed seed and an FDR threshold of 0.01 to identify high-confidence cells, and barcodes not meeting this threshold were excluded. Mitochondrial genes (prefixed with "mt-") were identified from feature metadata, and per-cell QC metrics—including total UMIs, number of detected genes, and percentage of mitochondrial reads—were calculated using scater::addPerCellQC() from the scater package (v1.30.1). Cells with fewer than 200 total UMIs or more than 7.5% mitochondrial content were removed, using thresholds described previously [28]. Principal component analysis on these QC metrics guided manual gating to select the final set of high-quality cells.

Single-cell RNA-seq Data Integration and Analysis

Five QC-filtered SingleCellExperiment objects were converted into Seurat objects (version 5.3.0) with sample identifiers ("p1"–"p5"). Log-normalized expression values were computed (log2(counts + 1)) and stored as sparse matrices. Individual Seurat objects were merged into a single combined object using sample-based cell identifiers.

The merged Seurat object was normalized using NormalizeData() with default parameters (LogNormalize, scale.factor = 10000), highly variable features were identified using FindVariableFeatures() with default settings (selection.method = "vst", nfeatures = 2000), and data were scaled using ScaleData() across those variable features with default settings. Principal component analysis (PCA) was performed on the top variable genes. An elbow plot of variance explained was used to select the first 20 PCs for downstream analyses. A shared nearest neighbor (SNN) graph was constructed (FindNeighbors) using these PCs, and clusters were detected at a resolution of 0.2 (FindClusters). Two-dimensional UMAP embeddings were computed (RunUMAP) for visualization, and plots were generated to display cells by sample and by cluster identity.

Supplementary Figures

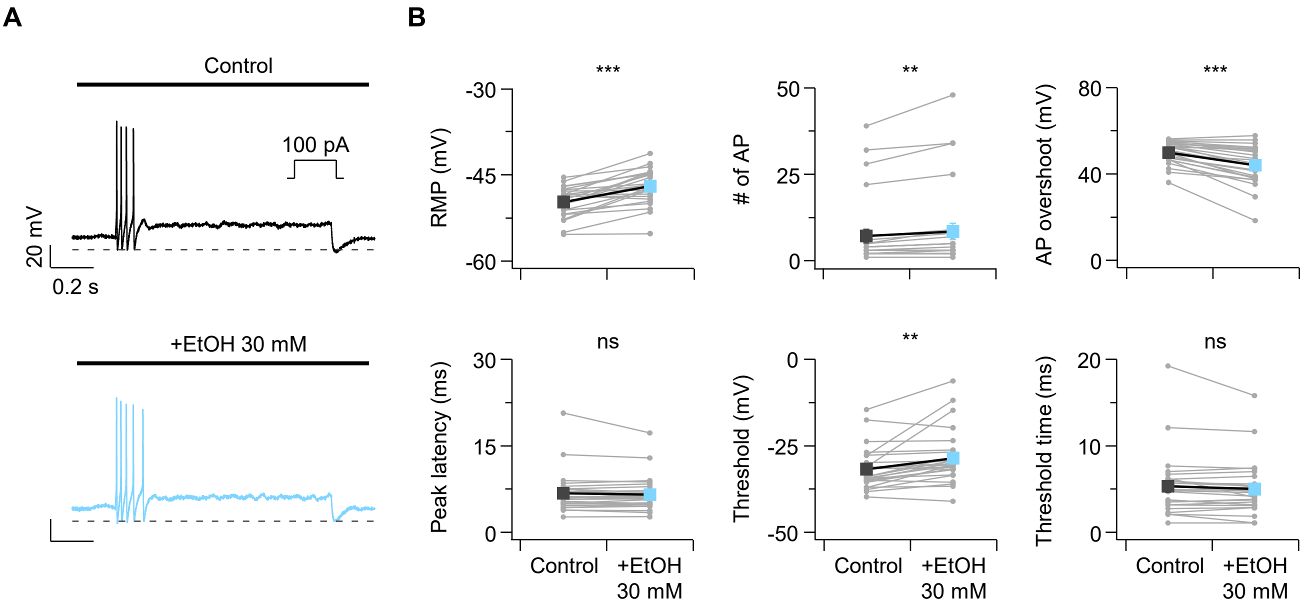

Figure S1. Effects of EtOH 30 mM on AP parameters in rat SCG neurons. (A) Representative AP traces before and after application of EtOH (30 mM). **(B)** Summary of changes in AP properties before and after treatment. Parameters shown from top to bottom: RMP (Ctrl-EtOH, n = 27, p = 1.3E^{-7}), number of AP (Ctrl-EtOH, n = 27, p = 0.00295), AP overshoot (Ctrl-EtOH, n = 27, p = 6.0E^{-7}), peak latency (Ctrl-EtOH, n = 27, p = 0.238), threshold (Ctrl-EtOH, n = 27, p = 0.00104), and threshold time (Ctrl-EtOH, n = 27, p = 0.058). Data are shown as mean ± SEM. Statistical significance was assessed using paired Student's *t*-test. ns, no significance; $^{**}p < 0.01$, $^{***}p < 0.001$.

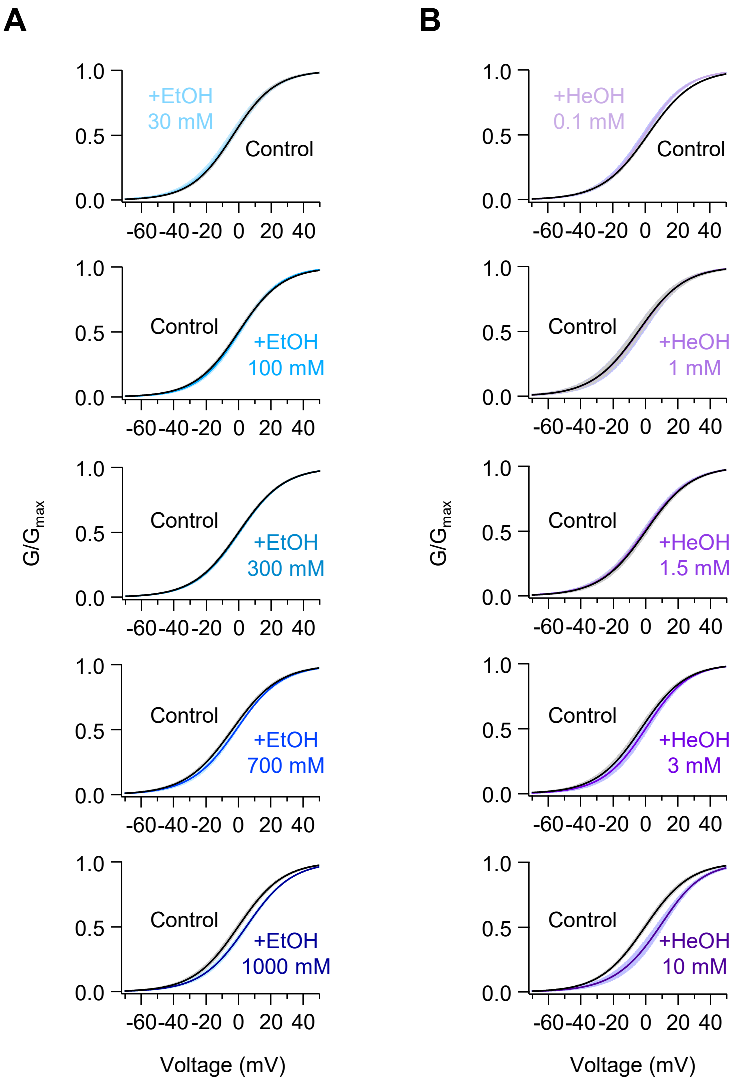

Figure S2. Effects of EtOH and HeOH on fitted activation curve.
Boltzmann fits of normalized conductance (G/G_{max}) curves recorded before and after treatment of **(A)** EtOH and **(B)** HeOH. $\Delta V_{1/2}$ (mV) after EtOH treatment (30, 100, 300, 700, 1000 mM): -0.5 ± 1.3, 0.5 ± 0.5, 0.4 ± 0.4, 2.3 ± 0.8, 5.0 ± 0.8 and $\Delta V_{1/2}$ (mV) after HeOH treatment (0.1, 1, 1.5, 3, 10 mM): -2.3 ± 0.7, 0.2 ± 0.7, -0.8 ± 0.8, 2.5 ± 0.8, 8.2 ± 1.8. Related to Figure 2.

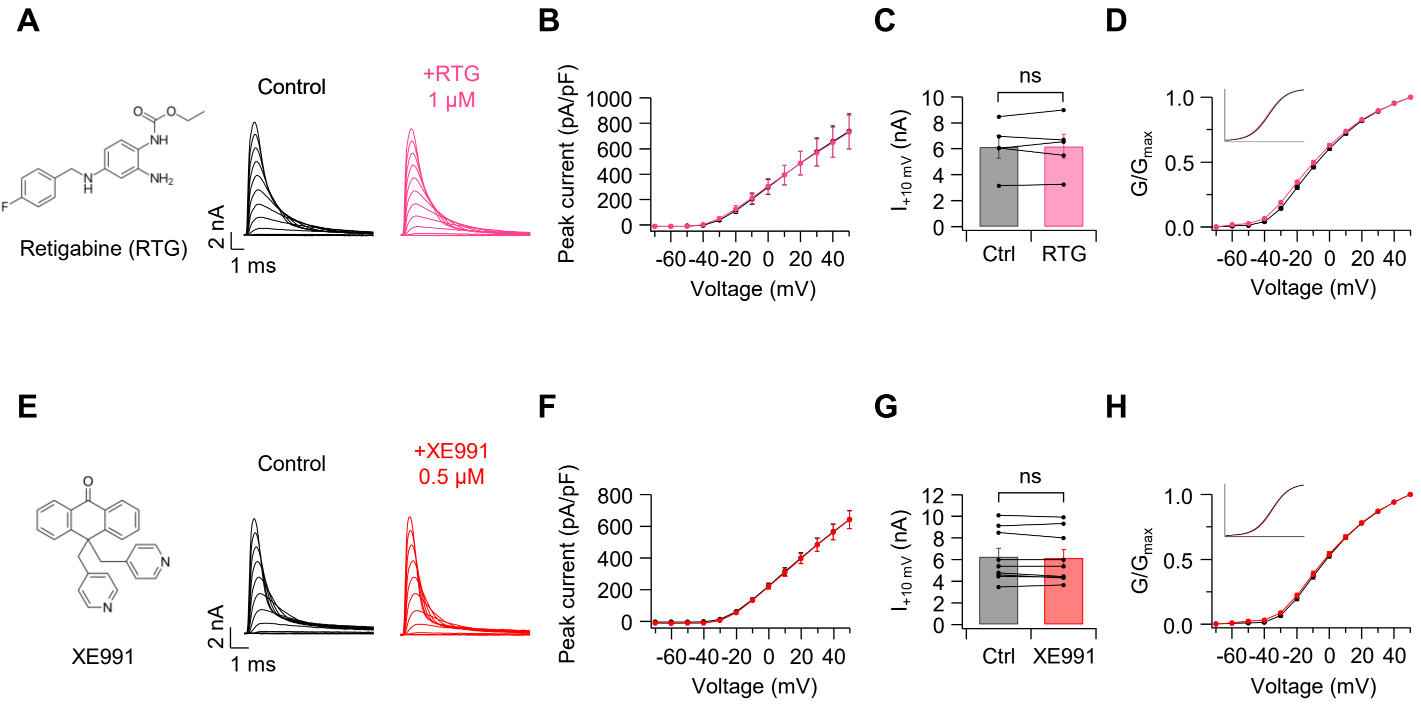

Figure S3. Sodium currents are unaffected by RTG and XE991 treatment.
(A) *Left*, chemical structure of RTG. *Right*, representative Na_V current traces before and after RTG treatment. **(B)** Peak current density at +10 mV before and after application of RTG 1 μM (n = 5). **(C)** Comparison of Na_V currents measured at +10 mV before and after treatment with 1 μM RTG (n = 5, p = 0.803). **(D)** Before and after treatment with RTG, conductance traces were normalized to G/G_{max}. The inset displays Boltzmann fit of the activation curve. $\Delta V_{1/2}$ (mV) after treatment of RTG 1 μM is 1.1 ± 0.4. **(E)** *Left*, the chemical structure of XE991. *Right*, representative Na_V current traces before and after XE991 treatment. **(F)** Peak current density at +10 mV before and after application of XE991 0.5 μM (n = 9). **(G)** Comparison of Na_V currents measured at +10 mV before and after treatment with 0.5 μM XE991 (n = 9, p = 0.204). **(H)** Before and after treatment with XE991, conductance traces were normalized to G/G_{max}. The inset displays Boltzmann fit of the activation curve. $\Delta V_{1/2}$ (mV) after treatment of XE991 0.5 μM is -0.7 ± 0.5. Data are shown as mean ± SEM. Statistical significance was assessed using paired Student's *t*-test. No significance is denoted as ns.

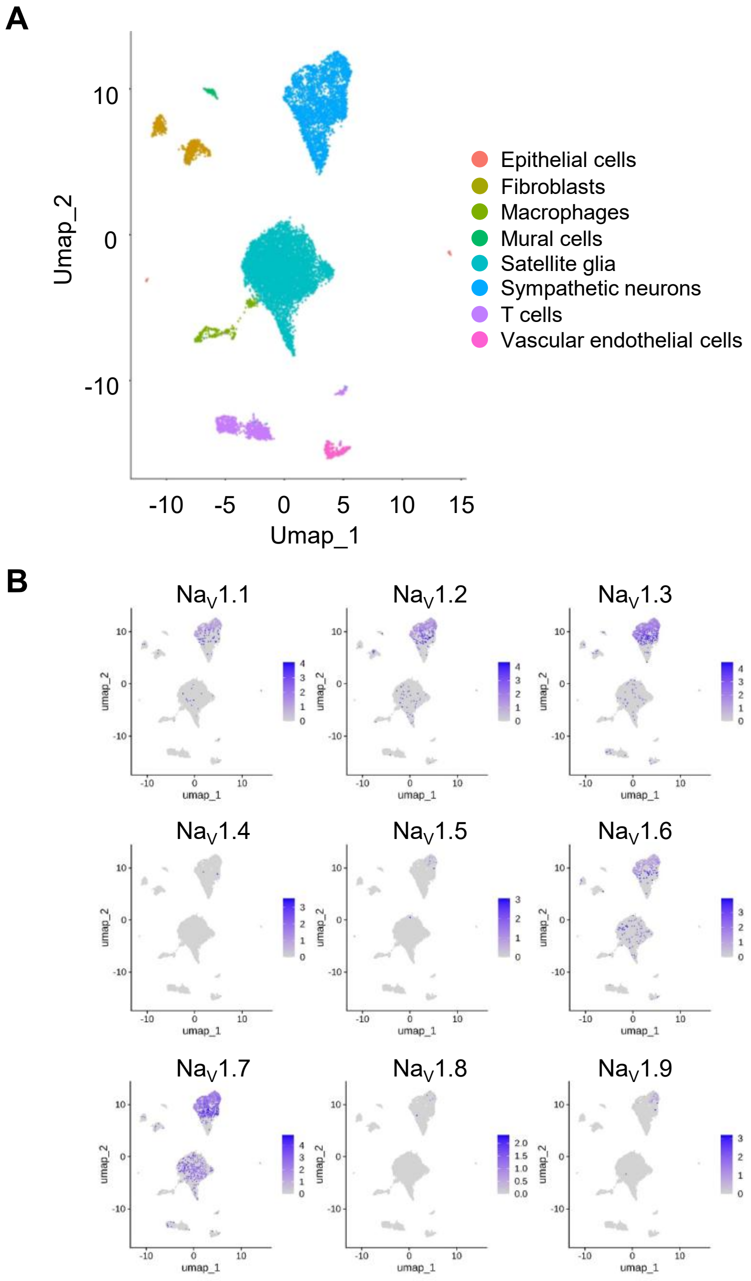

Figure S4. Expression of Na$_V$1.1–Na$_v$1.9 genes in each cell type in SCG.

(A) UMAP visualization showing cell types identified in adult mouse SCGs, with each cell type color-coded. **(B)** UMAP visualization depicting the expression levels of Na$_v$1.1–Na$_v$1.9 across cell types, represented as z-scores.

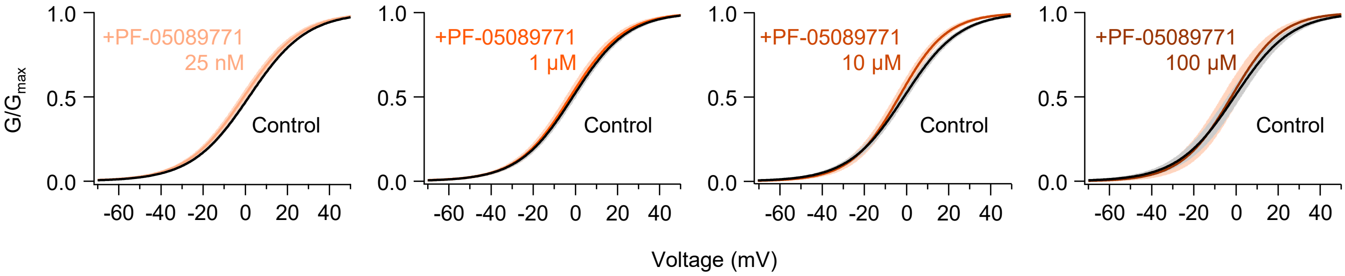

Figure S5. Effects of PF-05089771 on fitted activation curve.
Boltzmann fits of normalized conductance (G/G_{max}) curves recorded before and after treatment of PF-05089771. $\Delta V_{1/2}$ (mV) after PF-05089771 treatment (25 nM, 1, 10, 100 µM): -1.6 ± 1.3, -4.3 ± 3.7, 1.8 ± 2.6, -3.6 ± 0.2. Related to Figure 7.

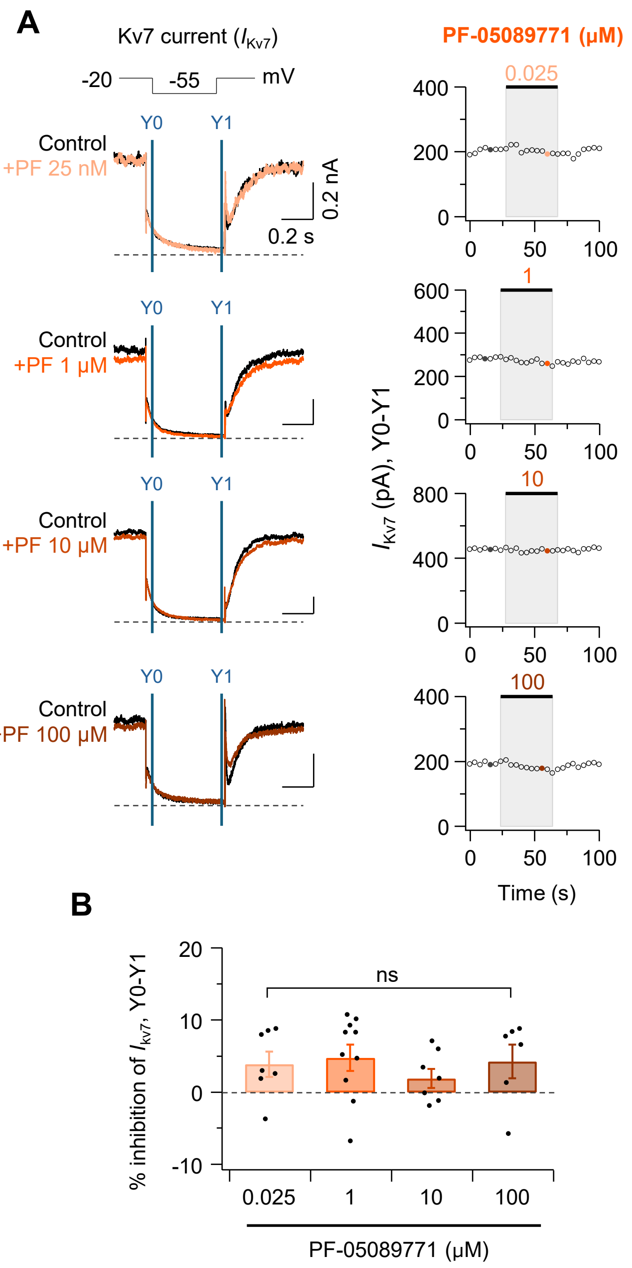

Figure S6. Effect of PF-05089771 on Kv7 channels in rat SCG neurons.
(A) *Left*, representative traces showing Kv7 currents recorded from primary cultured SCG neurons using a voltage protocol from −20 to −55 mV while the cells were incubated with PF-05089771. The dashed line indicates the zero-current level. *Right*, time-course analysis of I_{Kv7}(Y0−Y1) following sequential treatments of 0.025, 1, 10, and 100 μM PF-05089771. **(B)** Percent inhibition of Kv7 currents (Y0-Y1) after PF-05089771 treatment (0.025 μM, n = 7; 1 μM, n = 10; 10 μM, n = 7; 100 μM, n = 6; p = 0.705). Data are shown as mean ± SEM. Statistical significance was assessed using one-way ANOVA with Post hoc analysis using Tukey's HSD test. No significance is denoted as ns.

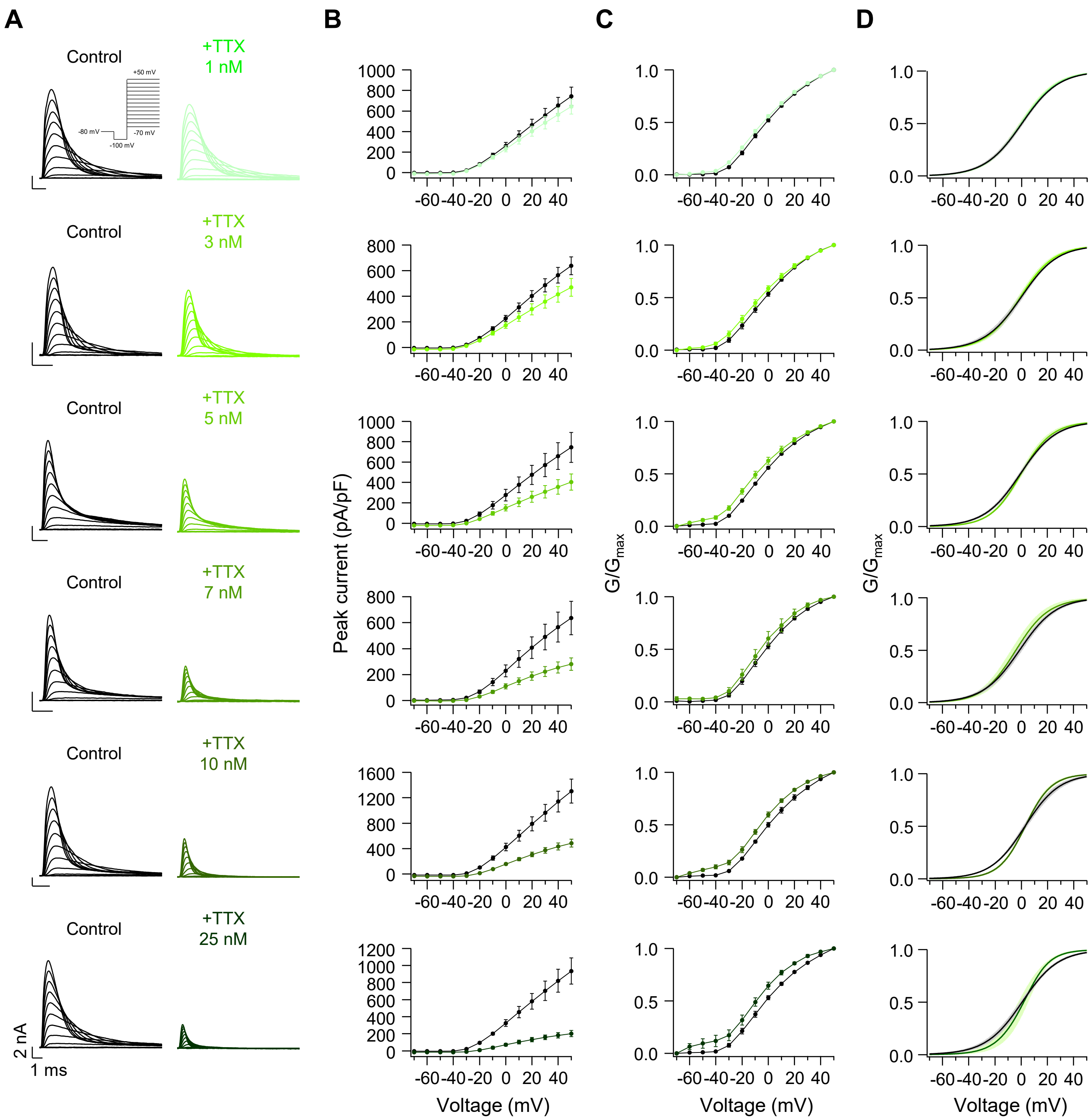

Figure S7. Dose-dependent inhibition of sodium currents by tetrodotoxin (TTX).
(**A**) Representative inverse gradient Na_V currents of SCG neurons before and after treatment with TTX. **(B)** Peak current density at +10 mV before and after TTX treatment (1 nM, *n* = 5; 3 nM, *n* = 7; 5 nM, *n* = 5; 7 nM, *n* = 2; 10 nM, *n* = 3; 25 nM, *n* = 3). Before and after treatment with TTX, conductance traces were normalized to G/G_{max}. **(C)** Raw traces and **(D)** Boltzmann fits. $\Delta V_{1/2}$ (mV) after TTX treatment (1, 3, 5, 7, 10, 25 nM): 0.4 ± 0.7, 0.5 ± 0.8, -0.7 ± 1.6, 2.3 ± 1.8, -0.07 ± 1.0, -0.7 ± 1.9.Data are shown as mean ± SEM.

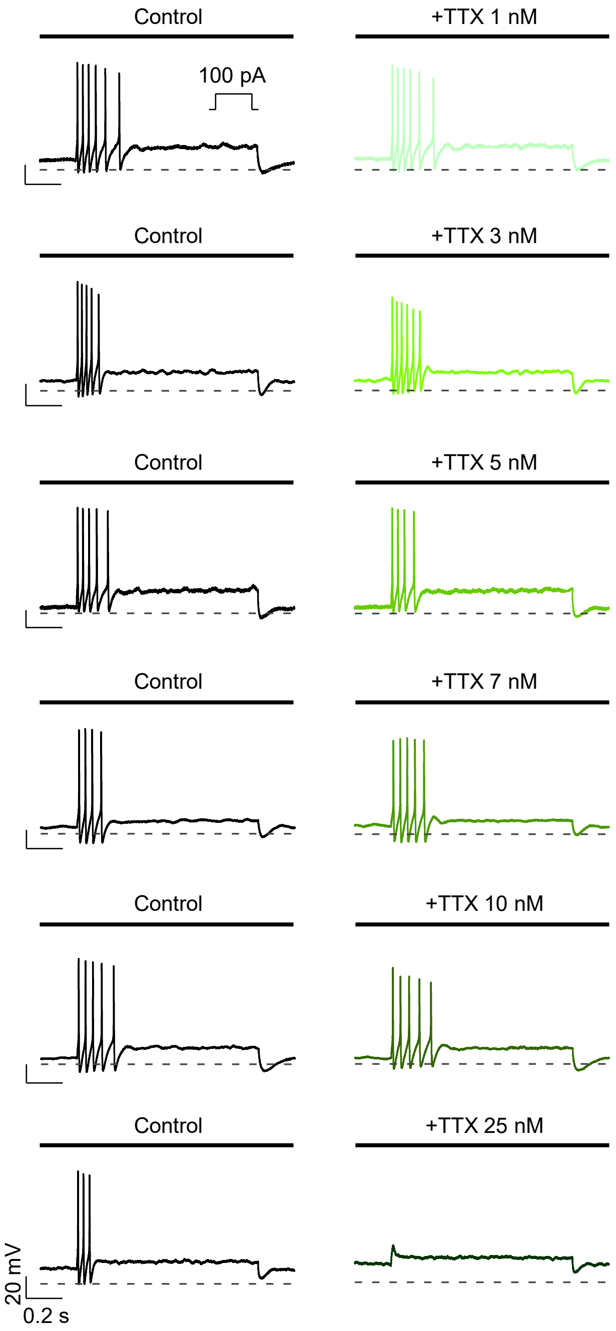

Figure S8. Effect of TTX on action potential firing.
Representative traces of action potential (AP) evoked by a 100 pA current injection in SCG neurons before and after treatment with TTX 1, 3, 5, 7, 10, and 25 nM.

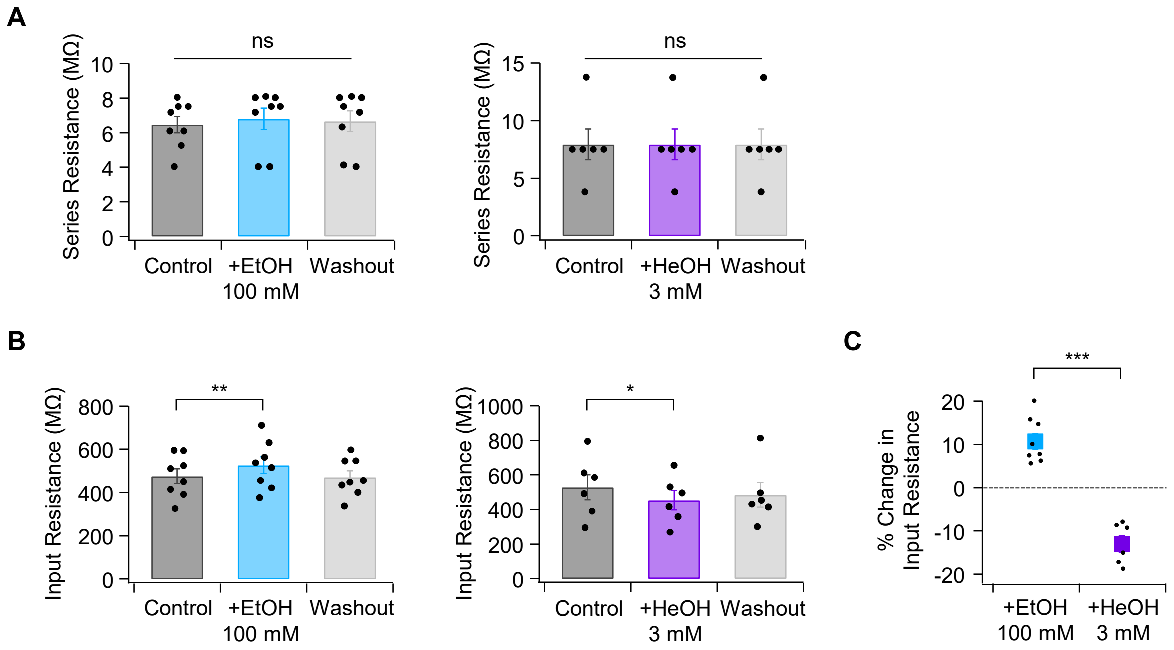

Figure S9. Recording stability and bidirectional modulation of input resistance by EtOH and HeOH.

(A) Series resistance remained stable throughout EtOH (Control, 6.5 ± 0.5; +EtOH 100 mM, 6.8 ± 0.6; Washout, 6.7 ± 0.6 MΩ, all $n = 8$) and HeOH experiments (Control, 7.9 ± 1.3; +HeOH 3 mM, 7.9 ± 1.3; Washout, 7.9 ± 1.3 MΩ, all $n = 6$), confirming maintained seal quality and recording stability. No significant difference were observed among EtOH and HeOH experiments (all $p > 0.6$). **(B)** Input resistance (R_{in}) showed bidirectional modulation; EtOH (Control, 476 ± 34; +EtOH 100 mM, 526 ± 39; Washout, 471 ± 31 MΩ, all $n = 8$) increased and HeOH (Control, 528 ± 72; +HeOH 3 mM, 454 ± 56; Washout, 485 ± 71 MΩ, all $n = 6$) decreased R_{in}. Control-EtOH, p = 0.0049; Control-Washout, p = 0.97; EtOH-Washout, p = 0.093 and Control-HeOH, p = 0.0232; Control-Washout, p = 0.37; HeOH-Washout, p = 0.59. **(C)** Summary of percent changes demonstrating opposing effects by EtOH (10.7 ± 1.9%) and HeOH (-13.1 ± 1.9%), $p = 1.6E^{-6}$. Data are shown as mean ± SEM. Statistical significance was assessed by repeated measures one-way ANOVA with Tukey's multiple comparisons test for (**A, B**) and unpaired Student's t-test for (**C**). ns, no significance; *$p < 0.05$, **$p < 0.01$, ***$p < 0.001$.

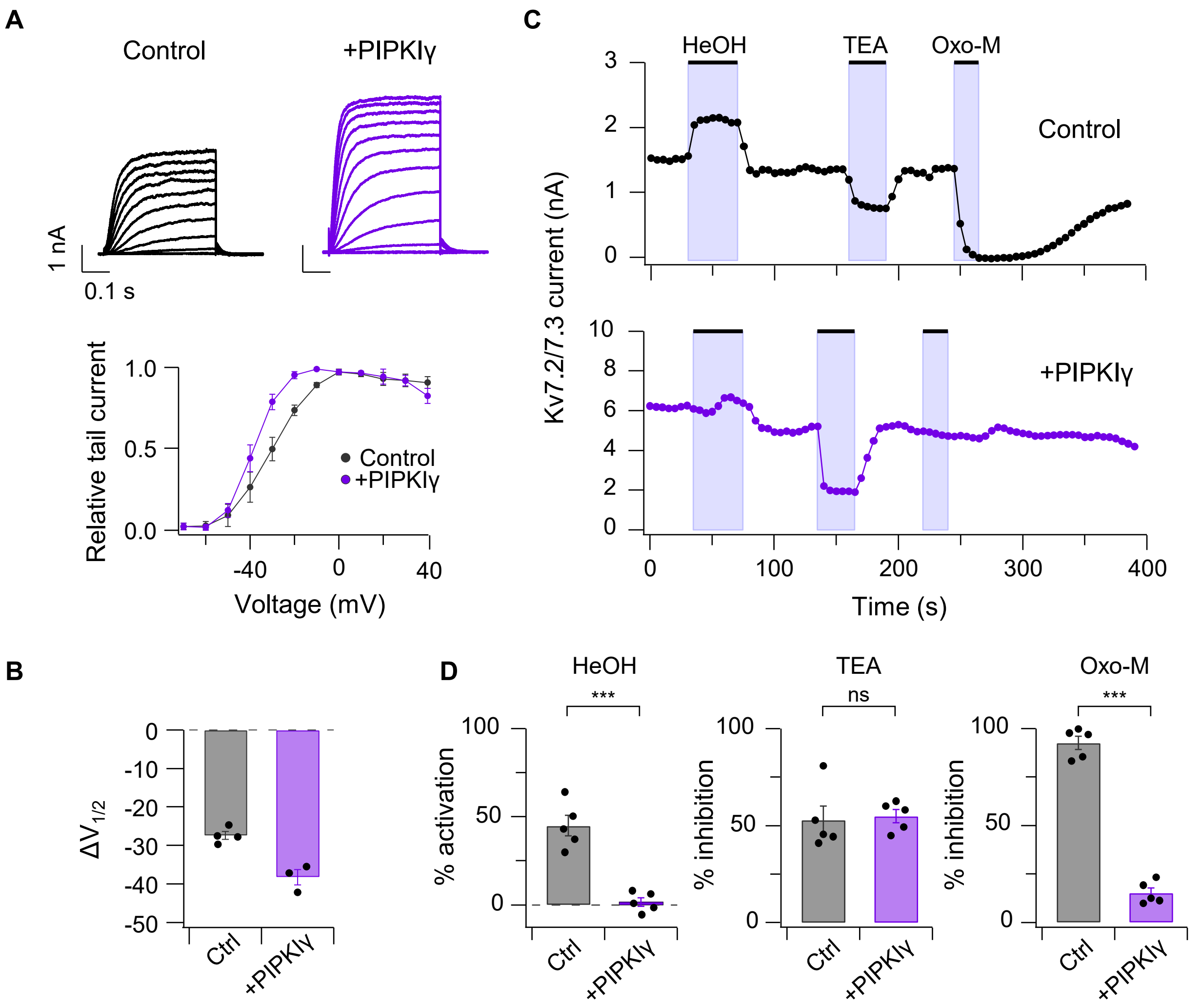

Figure S10. The stimulatory effect of HeOH on Kv7.2/7.3 currents is reduced by elevated plasma membrane PI(4,5)P_2.

(A) *Top*, Representative Kv7.2/7.3 currents in tsA201 cells transfected with Kv7.2/7.3 channels in control conditions (left) and with PIPKIγ overexpression (right). The cells were held at a holding potential of −80 mV, and depolarizing voltage steps were applied from −70 to +40 mV in 10 mV increments. *Bottom*, Voltage-activation curves showing normalized tail currents. **(B)** PIPKIγ overexpression induces a leftward shift in the voltage dependence of activation (Control: -27.4 ± 1.0 mV, n = 4; PIPKIγ overexpression: -38.3 ± 2.0 mV, n = 3). **(C)** Time-course recordings showing the effects of HeOH (10 mM), TEA (30 mM), and Oxo-M (10 μM) on Kv7.2/7.3 currents in control (top) and PIPKIγ-overexpressing cells (bottom). **(D)** Quantification of current modulation. PIPKIγ overexpression significantly reduces HeOH-induced activation (Control: 44.9 ± 5.9 mV, n = 5; PIPKIγ overexpression: 1.6 ± 2.5 mV, n = 5; p = 0.00014) and Oxo-M-induced inhibition (Control: 92.6 ± 3.4 mV, n = 5; PIPKIγ overexpression: 15.3 ± 2.6 mV, n = 5; p = 9.0E-8), while TEA-mediated pore block remains unaffected (Control: 52.8 ± 7.2 mV, n = 5; PIPKIγ overexpression: 54.9 ± 3.4 mV, n = 5; p = 0.17). Data are presented as mean ± standard error of the mean (SEM). Statistical significance was assessed using unpaired t-test. Statistical significance is denoted as ns, no significance; ***$p < 0.001$.
